# Supplementary material for: Solvent-pair surfactants enabled assembly of clusters and copolymers towards programmed mesoporous metal oxides
Source: Nat Commun. 2023 Dec 21;14:8493. doi: 10.1038/s41467-023-44193-z (PMC10739937; doi:10.1038/s41467-023-44193-z)
Supplement: Supplementary file 1 — Supplementary Information [file 41467_2023_44193_MOESM1_ESM.pdf]

Supplementary Information

Solvent-Pair Surfactants Enabled Assembly of Clusters and Copolymers towards Programmed Mesoporous Metal Oxides

Wenhe Xie[1,2], Yuan Ren[1], Fengluan Jiang[1], Xin-Yu Huang[1], Bingjie Yu[1], Jianhong Liu[1], Jichun Li[1], Keyu Chen[1], Yidong Zou[1], Bingwen Hu[3], Yonghui Deng[1,2]*

[1]Department of Chemistry, State Key Laboratory of Molecular Engineering of Polymers, Shanghai Key Laboratory of Molecular Catalysis and Innovative Materials, Collaborative Innovation Center of Chemistry for Energy Material (iChEM), Fudan University, Shanghai 200433, China

[2]State Key Lab of Transducer Technology, Shanghai Institute of Microsystem and Information Technology, Chinese Academy of Sciences, Shanghai 200050, China

[3]Shanghai Key Laboratory of Magnetic Resonance, State Key Laboratory of Precision Spectroscopy, School of Physics and Electronic Science, East China Normal University, Shanghai 200241, China

*e-mail: yhdeng@fudan.edu.cn;

Table of Contents

1. Supplementary experimental section (p. 3)
2. Homogeneity and heterogeneity of aqueous binary solvent (p. 9)
3. DFT calculations and molecular dynamics simulations revealing the origin of homogeneity and heterogeneity of aqueous binary solvent (p. 13)
4. FTIR, UV-vis and NMR spectra for the species co-existing with PEO-b-PS (p. 18)
5. Co-assembly of PEO-*b*-PS micelles and POMs (p. 23)
6. TG and XRD analysis (p. 26)
7. TEM characterization of $mK_2Mo_3O_{10}$ and $mLiV_3O_8$ (p. 29)
8. XPS and EDX element mapping of mMOs (p. 30)
9. SPEA enabled indirect co-assembly of POMs with other amphiphilic block copolymers (p. 36)
10. Co-assembly parameters (p. 37)

10.1 Optimizing PEO-*b*-PS: AMT ratio (p. 37)

10.2 Optimizing DMF: H_2O ratio (p. 38)

10.3 Two-step sequential thermal treatment process (p. 39)

11. Small angle X-ray scattering analysis (p. 40)
12. Adjustment of pore sizes and pore structures (p. 41)
13. Electron microscopy characterization of mWO_3 (p. 43)
14. Thermal stability of mN-WO_3 (p. 44)
15. Spectrum characterization of mN-WO_3 (p. 47)
16. X-ray absorption fine structure data of mN-WO_3 (p. 50)
17. DFT calculations and acetone sensing tests of mN-WO_3 based gas sensor (p. 51)
18. Co-assembly between AMT and other POMs (p. 62)
19. Co-assembly between AMT and molecular metal salts (p. 66)

1. Supplementary experimental section

Chemicals and Materials

Phosphotungstic acid ($H_3PW_{12}O_{40}·xH_2O$, analytical reagent (AR)), phosphomolybdic acid hydrate ($H_3PMo_{12}O_{40}$, AR), ammonium molybdate tetrahydrate ($(NH_4)_6Mo_7O_{24}·4H_2O$, 99.9% metals basis), ammonium metavanadate (NH_4VO_3, AR), potassium metavanadate (KVO_3, 99.9% metals basis), iron (III) chloride hexahydrate ($FeCl_3·6H_2O$, 98%), aluminum chloride hexahydrate ($AlCl_3·6H_2O$, 97%), erbium trinitrate pentahydrate ($Er(NO_3)_3·5H_2O$, 99.9% metals basis), cerium chloride ($CeCl_3$, 99.9% metals basis), chloroplatinic acid hexahydrate ($H_2PtCl_6·6H_2O$, AR), europium nitrate hexahydrate ($Eu(NO_3)_3·6H_2O$, 99.9% metals basis), cuprous bromide (CuBr, 99.9%), 2-bromoisobutyryl bromide ($C_4H_6Br_2O$, 98%) were purchased from Aladdin. Sodium phosphotungstate ($Na_3PW_{12}O_{40}·xH_2O$, AR), hydrochloric acid (HCl, 36.0~38.0%), nickel (II) chloride hexahydrate ($NiCl_2·6H_2O$, AR), cobalt (II) chloride hexahydrate ($CoCl_2·6H_2O$) were purchased from Sino-Pharm Chemical Reagent Co. Ltd. Potassium molybdate (K_2MoO_4, 99%), trisodium tetraoxovanadate dodecahydrate ($Na_3VO_4·12H_2O$, anhydrous basis) were purchased from Macklin. Sodium metatungstate monohydrate ($Na_6H_2W_{12}O_{40}·H_2O$), lithium vanadium oxide ($LiVO_3$, 99.9% metals basis) were purchased from Alfa Aesar. Silicomolybdic acid solution was purchased from Aldrich.

Synthesis of PEO-b-PS diblock copolymers

The PEO-*b*-PS was prepared by a simple ATRP method. 20.0 g of monomethoxy PEO (M_w = 5000) was dissolved in 120 ml of THF and 20 ml of pyridine. The

solution was cooled in ice-water bath. Then, 3.3 ml of 2-bromoisobutyryl bromide was added dropwise under stirring for 30 min. The resultant solution was further stirred at 30 °C for 24 h. The white product of PEO-Br was precipitated by dropping the reaction solution in cold ether and further dried in vacuum. 5.0 g of PEO-Br, 0.15 g of CuBr, 0.6 ml of PMDETA and 40 ml of styrene were added to an ampoules bottle and fully degassed with three freeze-pump-thaw cycles. It was subsequently immersed in an oil bath at 115 °C under stirring. The reaction continued for 2-6 h to control the polymerization degree of styrene, after which the system was diluted by 100 ml of THF and filtered through Al_2O_3 column to remove Cu complexes. 400 ml of petroleum ether was poured into the solution to precipitate PEO-*b*-PS product. The product was dried in vacuum.

Synthesis of ordered mesoporous phosphorus-doped WO_3 (mP-WO_3)

mP-WO_3 was synthesized following the same method as that for the mN-WO_3 except that $H_3PW_{12}O_{40}$ was used as the precursor. The mass ratio of PEO-*b*-PS/POMs was 1:3. The thermal treatment procedure was calcination at 550 °C for 0.5 h in N_2 (heating rate, 1 °C/min below 350 °C and 5 °C/min above 350 °C) and then at 400 °C for 0.5 h in air (5 °C/min).

Synthesis of ordered mesoporous $Na_2W_2O_7$&WO_3 (m$Na_2W_2O_7$&WO_3)

m$Na_2W_2O_7$&WO_3 was synthesized following a similar method with mN-WO_3 except that $Na_6H_2W_{12}O_{40}$ was used as the precursor. The mass ratio of PEO-*b*-PS/POMs was 1:4. The thermal treatment procedure was calcination at 500 °C for 0.5 h in N_2 (heating rate, 1 °C/min below 350 °C and 5 °C/min above 350 °C) and then at 400 °C

for 0.5 h in air (5 °C/min).

Synthesis of ordered mesoporous phosphorus-doped $Na_2W_2O_7$&WO_3 (mP-$Na_2W_2O_7$&WO_3)

mP-$Na_2W_2O_7$&WO_3 was synthesized following a similar method with mN-WO_3 except that $Na_3PW_{12}O_{40}$ was used as the precursor. The mass ratio of PEO-*b*-PS/POMs was 1:3. The thermal treatment procedure was calcination at 450 °C for 0.5 h in N_2 (heating rate, 1 °C/min below 350 °C and 5 °C/min above 350 °C) and then at 400 °C for 0.5 h in air (5 °C/min).

Synthesis of ordered mesoporous nitrogen-doped MoO_3 (mN-MoO_3)

mN-MoO_3 was synthesized following a similar method with mN-WO_3 except that $(NH_4)_6Mo_7O_{24}$ was used as the precursor and PEO_{114}-*b*-PS_{275} (M_w = 33610 g/mol, polydispersity index = 1.44) was used as the template. The mass ratio of PEO-*b*-PS/POMs was 1:2. The thermal treatment procedure was calcination at 350 °C for 3 h in N_2 (heating rate, 1 °C/min) and then at 400 °C for 15 min in air (5 °C/min).

Synthesis of ordered mesoporous phosphorus-doped MoO_3 (mP-MoO_3)

mP-MoO_3 was synthesized following a similar method with mN-WO_3 except that $H_3PMo_{12}O_{40}$ was used as the precursor. The mass ratio of PEO-*b*-PS/POMs was 1:3 and light-yellow colloidal solution was formed after mixing. The thermal treatment procedure was calcination at 450 °C for 0.5 h in N_2 (heating rate, 1 °C/min below 350 °C and 5 °C/min above 350 °C) and then at 350 °C for 1 h in air (5 °C/min).

Synthesis of ordered mesoporous silicon-doped MoO_3 (mSi-MoO_3)

mSi-MoO_3 was synthesized following a similar method with mN-WO_3 except that

$H_4SiMo_{12}O_{40}$ was used as the precursor and PEO$_{114}$-*b*-PS$_{275}$ (M_w = 33610 g mol^{-1}, polydispersity index = 1.44) was used as the template. The mass ratio of PEO-*b*-PS/POMs was 1:4 and light-yellow colloidal solution was formed after mixing. The thermal treatment procedure was calcination at 400 °C for 1 h in N_2 (heating rate, 1 °C/min below 350 °C and 5 °C/min above 350 °C) and then at 350 °C for 1 h in air (5 °C/min).

Synthesis of ordered mesoporous vanadium-based metal oxides (mN-V_2O_5, mNaV_3O_8, m$K_3V_5O_{14}$, mLiV_3O_8) and m$K_2Mo_3O_{10}$

0.01 g of amphiphilic diblock copolymer PEO$_{114}$-*b*-PS$_{110}$ (M_w = 16450 g/mol, polydispersity index = 1.05) was dissolved in 9 mL of DMF and followed by dropping 0.5 mL of water. Then, 0.04 g of the precursors (e.x., NH_4VO_3, Na_3VO_4, KVO_3, $LiVO_3$) were added to the solution to form a suspension. The mass ratio of PEO-*b*-PS/precursors was 1:4. After 50 μL hydrochloric acid aqueous solution was added (6 mol/L), the colorless suspension gradually turns into yellow, and finally becomes orange colloidal solution. m$K_2Mo_3O_{10}$ was synthesized following a similar method with vanadium-based metal oxides except that K_2MoO_4 was used as the precursor and the mass ratio of PEO-*b*-PS/POMs was 1:3. Yellow colloidal solution was formed after the addition of hydrochloride acid. After stirring for 2 h, the colloidal solution was cast onto glass Petri dished to evaporate DMF and water at 40 °C for 24 h, followed by further heating in an oven at 100 °C for 24 h to solidify the structure. Vanadium-based mMOs usually crystallize rapidly in air and they are difficult to crystallize in inert atmosphere. Therefore, it is necessary to precisely

control the calcination time in air. To obtain the highly ordered mesoporous metal oxides, the as-made PEO-*b*-PS/POMs were calcined at 350 °C for 3 h in N_2 (heating rate, 1 °C/min) and then at 400 °C for 10 min in air (5 °C/min). To obtain the highly crystallized mesoporous metal oxides, the calcination time in air was prolonged to 30 min. The pure mMOs were obtained by washing the precipitated chlorate (e.x., NaCl, KCl) with deionized water.

Synthesis of mesoporous N-WO_3/MoO_3 composite

mN-WO_3/MoO_3 was synthesized following a same method as that for the mN-WO_3 except that the mixture of $(NH_4)_4W_{12}O_{48}$ and $(NH_4)_6Mo_7O_{24}$ was used as the precursor. The mass ratio of $(NH_4)_4W_{12}O_{48}$/$(NH_4)_6Mo_7O_{24}$ was 30:1 (4.5 mol% MoO_3), 14:1 (9 mol% MoO_3), 10:1 (12.3 mol% MoO_3), 5:1 (22 mol% MoO_3) and 2:1 (41.2 mol% MoO_3). The mass ratio of PEO-*b*-PS/POMs was 1:3. The thermal treatment procedure was calcination at 500 °C for 0.5 h in N_2 (heating rate, 1 °C/min below 350 °C and 5 °C/min above 350 °C) and then at 400 °C for 0.5 h in air (5 °C/min).

Synthesis of heteroatom-doped mesoporous N-WO_3

0.05 g of PEO_{114}-*b*-PS_{200} (M_w = 25800 g/mol, polydispersity index = 1.08) was dissolved in 4.5 mL of DMF to form a homogeneous solution. Meanwhile, 0.15 g of $(NH_4)_6H_2W_{12}O_{40}$ was dissolved in 0.5 mL of water. Gradually adding the $(NH_4)_6H_2W_{12}O_{40}$ water solution to copolymer DMF solution with a high-speed mixer, a light-blue colloidal solution was obtained. Then, 0.005 g molecular salt ($FeCl_3{\cdot}6H_2O$, $AlCl_3{\cdot}6H_2O$, $Er(NO_3)_3{\cdot}5H_2O$, $NiCl_2{\cdot}6H_2O$, $CeCl_3$, $Eu(NO_3)_3$, $Co(CH_3COO)_2{\cdot}4H_2O$, $Cu(CH_3COO_2)_2$, $Cr(NO_3)9{\cdot}H_2O$) was added to above solution. For the synthesis of

Fe-Co-Ni-Cr-Al co-doped mN-WO_3, molecular salts were added in sequence and each of them was 0.005 g. Subsequent procedures were same as that for the mN-WO_3.

Synthesis of Pt-loaded mesoporous N-WO₃

0.05 g of PEO_{114}-*b*-PS_{200} (M_w = 25800 g/mol, polydispersity index = 1.08) was dissolved in 4.5 mL of DMF to form a homogeneous solution. Meanwhile, 0.15 g of $(NH_4)_6H_2W_{12}O_{40}$ was dissolved in 0.5 mL of water. Gradually adding the $(NH_4)_6H_2W_{12}O_{40}$ water solution to copolymer DMF solution with a high-speed mixer, a light-blue colloidal solution was obtained. Then, 0.015 g $HPtCl_6{\cdot}6H_2O$ was added to above solution to form a pale yellow colloidal solution. Subsequent procedures were same as that for the mN-WO_3.

2. Homogeneity and heterogeneity of aqueous binary solvent

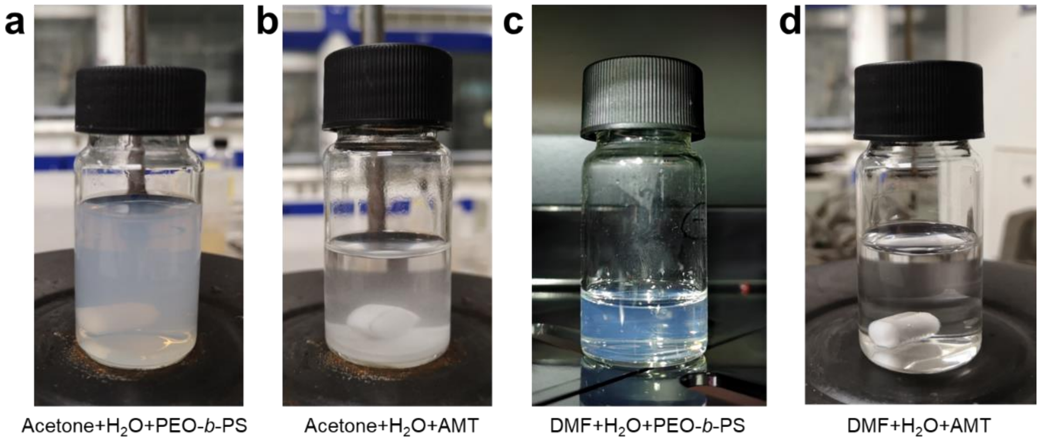

Supplementary Fig. 1. Optical photographs of PEO-*b*-PS/acetone/H_2O (a), AMT/acetone/H_2O (b), PEO-*b*-PS/DMF/H_2O (c) and AMT/DMF/H_2O (d).

Non-acidic POMs, such as ammonium metatungstate (AMT), are usually insoluble in organic solvents. However, it can be stably dissolved in some aqueous binary solvents. **Supplementary Fig. 1** shows the dissolution of PEO-*b*-PS and AMT in DMF/H_2O (10 vol%) and acetone/H_2O (10 vol%), respectively. DMF and acetone are good solvents for PEO-*b*-PS. With the introduction of 10% H_2O into the DMF/PEO-*b*-PS or acetone/PEO-*b*-PS solution, PEO-*b*-PS molecules underwent self-assembly to form colloidal micelles because water is poor solvent for PS segment (Figure S1a and c). Dropping AMT aqueous solution (0.4 g AMT in 1 mL H_2O) to 9 mL organic solvent under magnetic stirring, AMT can be readily dissolved in DMF/H_2O (Figure S1d) but it precipitates in acetone/H_2O binary solution (Figure S1b).

We further investigate the solubility of AMT in other organics/H_2O binary solvent. Dropping AMT aqueous solution (0.6 g AMT in 0.5 mL H_2O) to 4.5 mL organic solvent under magnetic stirring, AMT can be readily dissolved in aqueous

binary solvent systems with DMF, N, N-dimethylacetamide (DMA), dimethyl sulfoxide (DMSO), pyridine, N-methylpyrrolidone, N, N-dimethylpropanamide, N, N-diethylformamide. But it precipitates in aqueous binary solvent systems with THF, 1, 4-dioxane, acetone and ethanol. Therefore, aqueous binary solvents with homogeneity at the molecular level can be used to dissolve non-acidic POMs.

Supplementary Table 1. Three types of organic solvents which can dissolve amphiphilic copolymers

	Type-I	Type-II	Type-III
H_2O	√	√	×
AMT	×	×	×
AMT (aq.)	√	×	×
Type-I	DMF, DMA, DMSO, pyridine, N-methylpyrrolidone, N, N-dimethylpropanamide, N, N-diethylformamide, etc.		
Type-II	THF, 1, 4-dioxane, acetone, etc.		
Type-III	ethyl acetate, chloroform, etc.		

"√" means the two species are miscible. "×" means phase separation between the two species.

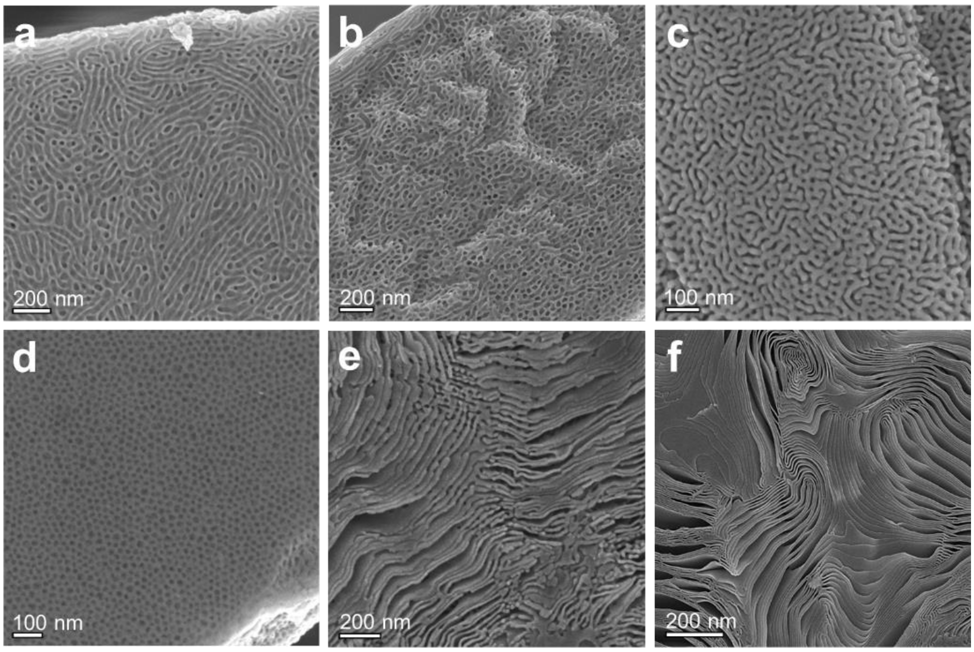

Supplementary Fig. 2. SEM images of N-WO_3 mesostructures synthesized using AMT and PEO_{114}-*b*-PS_{200} in different aqueous binary solvent. (a) DMA/H_2O. (b) N, N-dimethylpropanamide/H_2O. (c) Pyridine/H_2O. (d) N-methylpyrrolidone/H_2O. (e) DMSO/H_2O. (f) N, N-diethylformamide/H_2O.

3. DFT calculations and molecular dynamics simulations revealing the origin of homogeneity and heterogeneity of aqueous binary solvent

DFT calculations:

The DFT calculations were performed through the Gaussian 16 package[1]. Geometrical optimization was implemented using the M06-2X-D3 method[2] with aug–cc-pvtz basis set. Moreover, the electrostatic potential (ESP) was calculated based on Multiwfn (Version 3.7)[3-5] and visualized by VMD software (Version 1.9.3)[6]. The counterpoise correction energy term was obtained using the Boys and Bernardy method.

Molecular dynamics simulations:

MD simulations were performed using Gromacs 2019.4 package.[7,8] General Amber force fields parameters[9] and RESP charges[10] were used and generated by the ANTECHAMBER program in AmberTools for THF molecules.[11] The force field for DMF was taken from previous publications.[12] TIP4P water model was used to describe water molecules. The geometrical optimizations of $Na_3PW_{12}O_{40}$ were carried out using the Hartree-Fock method. The double valence 6-31g* basis set and the effective core potential LanL2DZ basis sets were respectively assigned to nonmetal elements (P, O) and W elements. The Mulliken charge distribution was used to describe $Na_3PW_{12}O_{40}$ in force field. The details of the calculated systems are listed in Supplementary Table 4. To obtain the equilibrium structure of each simulation system, 50 ns long NPT runs were conducted. Visualization of the structures was made by using VWD software.[6]

Supplementary Table. 2. Dipole moment values of different molecules

Molecule	Dipole moment/D
Dimethyl sulfoxide (DMSO)	3.96
N, N-dimethylformamide (DMF)	3.82
N, N-dimethylacetamide (DMA)	3.7
N, N-dimethylpropanamide	N/A
N, N-diethylformamide	N/A
N-methylpyrrolidone	N/A
Acetone	2.88
Pyridine	2.215
Water	1.8546
Ethyl acetate	1.78
Tetrahydrofuran	1.75
Ethanol	1.70
Chloroform	N/A
1, 4-dioxane	None

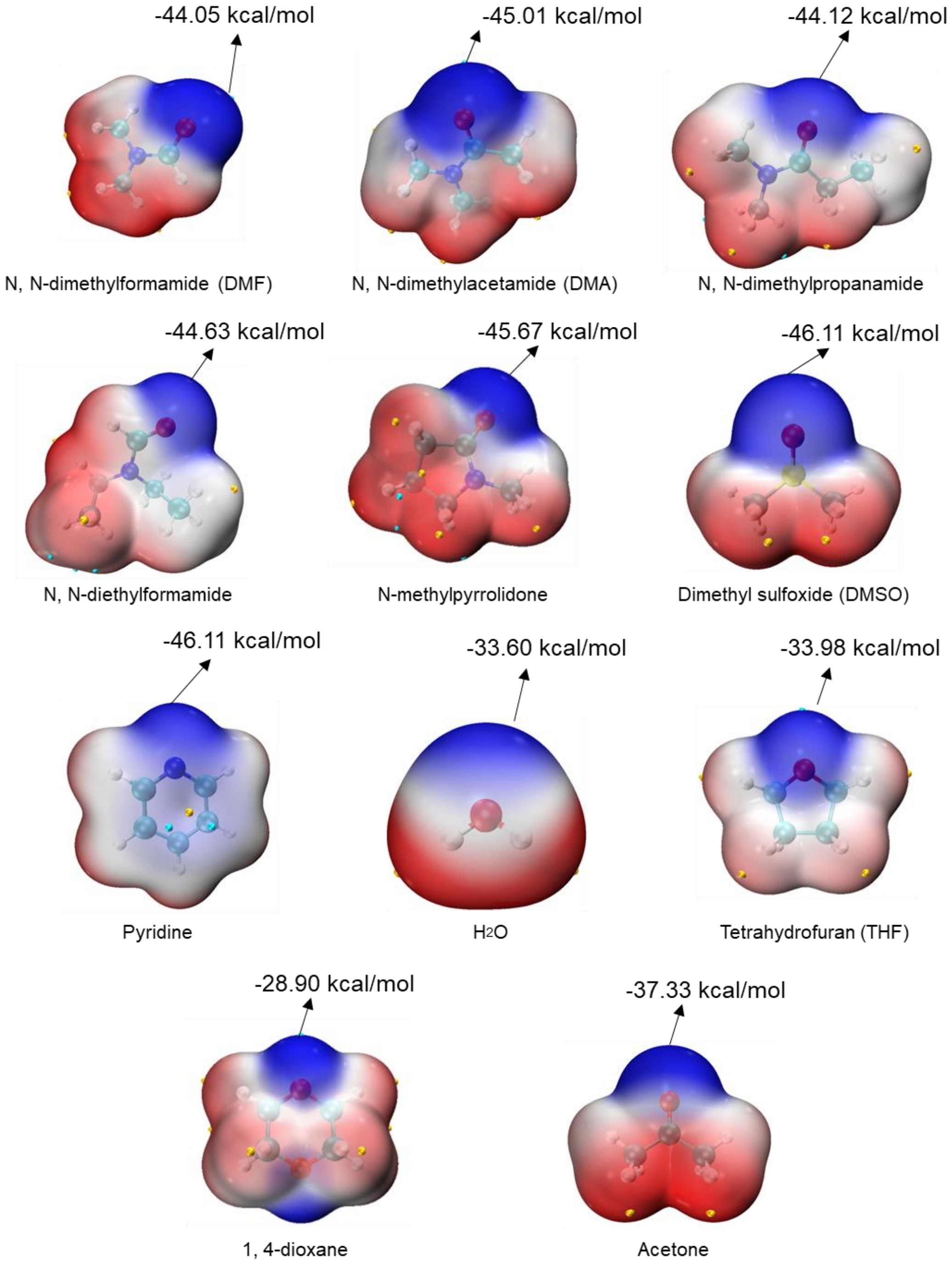

Supplementary Fig. 3. Quantitative molecular electrostatic surface potential values in kcal/mol mapped on corresponding 0.001 a.u. electron density isosurface of different molecules. Regions with the most negative (Vs, min) and most positive (Vs, max) electrostatic surface potentials are colored red and blue, respectively.

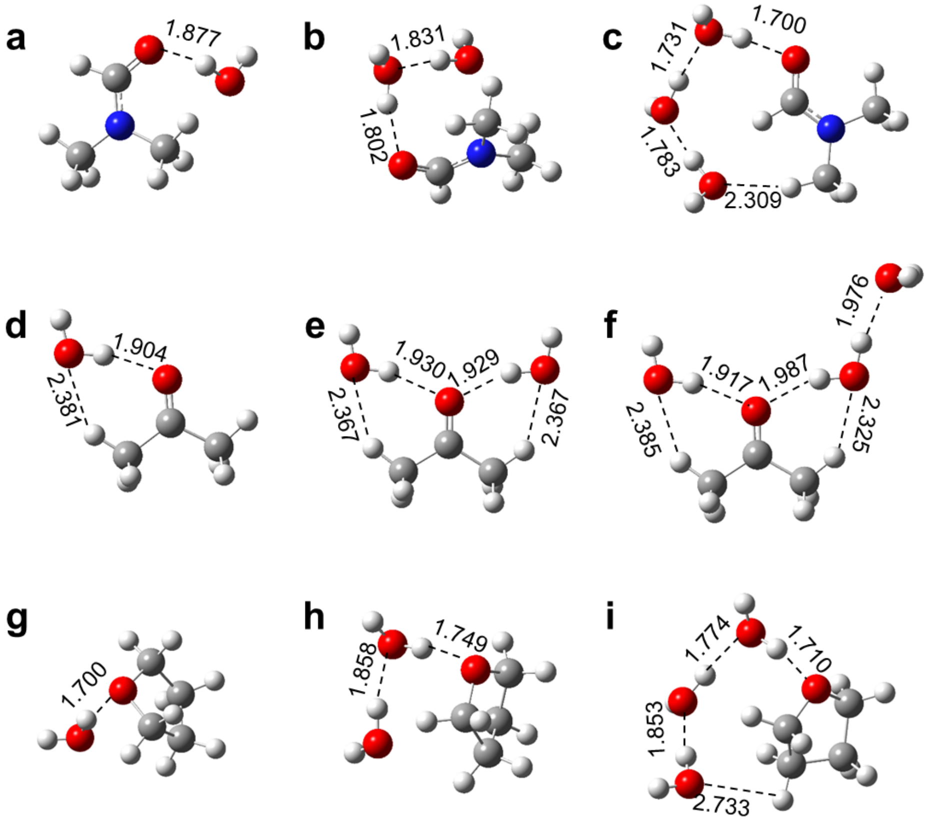

Supplementary Fig. 4. Optimized geometries for (a-c) DMF-$(H_2O)_n$, (d-f) Acetone-$(H_2O)_n$ and (g-i) THF-$(H_2O)_n$ complexes with n = 1-3. The values labeled are intermolecular bond lengths.

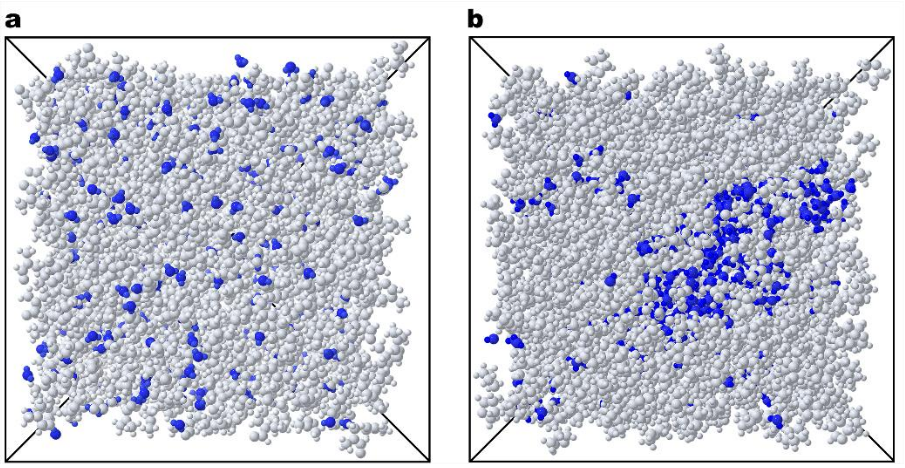

Supplementary Fig. 5. Snapshots of MD trajectories at 50 ns for DMF-water system (a) and THF-water system (b).

Supplementary Table 3. Selected physical properties of the minimum energy geometries of DMF-$(H_2O)_n$, Acetone-$(H_2O)_n$ and THF-$(H_2O)_n$ (n = 1-3) complexes obtained using M06-2X-D3/aug-cc-pVTZ method

**Molecule**	**C-O**	**C-H**	**C-O…H**	**H…O-H**	**E_{int} (kcal/mol)**
DMF	1.210	1.101	-	-	-
DMF-H_2O	1.220	1.100	1.877	-	-7.73
DMF-$(H_2O)_2$	1.225	1.099	1.802	1.831	-17.85
DMF-$(H_2O)_3$	1.229	1.097	1.700	1.731/1.783	-27.95

Molecule	C-O	C-H…O	C-O…H	H…O-H	E_{int} (kcal/mol)
Acetone	1.205		-	-	-
Acetone-H_2O	1.211	2.381	1.904	-	-6.98
Acetone-$(H_2O)_2$	1.217	2.367/2.367	1.930/1.929	-	-13.24
Acetone-$(H_2O)_3$	1.215	2.385/2.325	1.917/1.987	1.976	-17.52

Molecule	C-O	C-O…H	H…O-H	E_{int} (kcal/mol)
THF	1.417/1.417	-	-	-
THF-H_2O	1.424/1.424	1.886	-	-7.20
THF-$(H_2O)_2$	1.440/1.428	1.749	1.858	-16.33
THF-$(H_2O)_3$	1.440/1.436	1.710	1.774/1.853	-24.37

Supplementary Table 4. Molecular Dynamics (MD) simulation details for the DMF-H_2O (S1), THF-H_2O (S2) and DMF-H_2O-POMs (S3) systems

	S1	S2	S3
No. DMF in box	1000	-	418
No. Water in box	500	500	40
No. THF in box	-	1000	-
No. $Na_3PW_{12}O_{40}$ in box	-	-	1

4. FTIR, UV-vis and NMR spectra for the species co-existing with PEO-*b*-PS

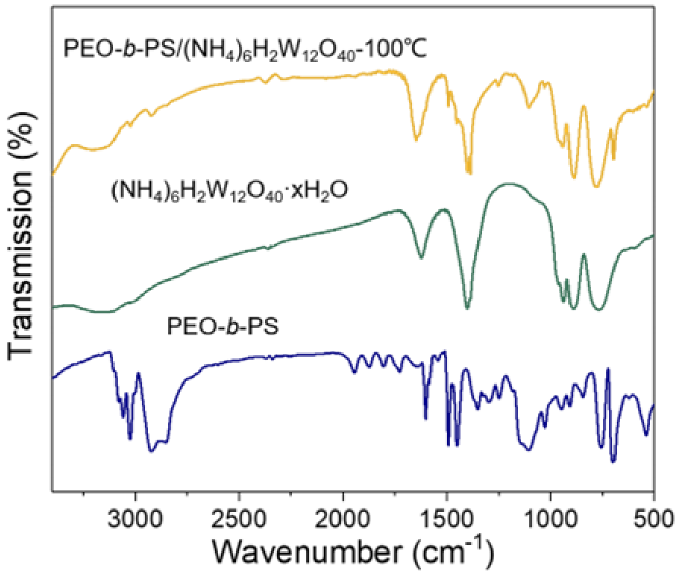

Supplementary Fig. 6. FTIR spectra of PEO-*b*-PS, $(NH_4)_6H_2W_{12}O_{40}\cdot xH_2O$ and PEO-*b*-PS/$(NH_4)_6H_2W_{12}O_{40}$ composites after thermal treatment at 100 °C to remove the solvents.

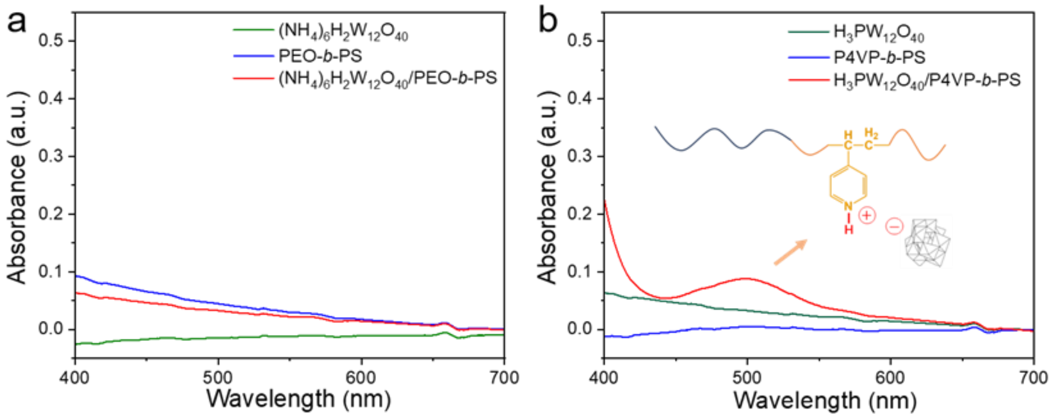

Supplementary Fig. 7. UV-vis absorption spectra of PEO-*b*-PS, $(NH_4)_6H_2W_{12}O_{40}$, PEO-*b*-PS/$(NH_4)_6H_2W_{12}O_{40}$ (a) and P4VP-*b*-PS, $H_3PW_{12}O_{40}$, P4VP-*b*-PS/$H_3PW_{12}O_{40}$ (b) in DMF/H_2O (9:1 v/v).

FTIR shows that the spectrum of PEO-*b*-PS/$(NH_4)_6H_2W_{12}O_{40}$ composite is a simple additive combination of the spectra of PEO-*b*-PS and $(NH_4)_6H_2W_{12}O_{40}$ (**Supplementary Fig. 6**). New characteristic peaks and significant shift of peak position were not found, implying that no new bonds or strong intermolecular

interactions were formed (e.g., hydrogen bond and coordination bond).

In order to provide more evidence for the proposed SPEA process requiring no interactions between polymers and non-acidic POMs, we employed UV-vis spectroscopy to study whether PEO-*b*-PS has direct strong intermolecular interactions with $(NH_4)_6H_2W_{12}O_{40}$ or not. We performed the UV-vis analysis by mixing the PEO-*b*-PS and $(NH_4)_6H_2W_{12}O_{40}$ in DMF/H_2O (9:1 v/v) according to typical experimental conditions in our study. The UV-vis spectra reveal that PEO-*b*-PS/DMF/H_2O, $(NH_4)_6H_2W_{12}O_{40}$/DMF/H_2O, and the mixed PEO-*b*-PS/$(NH_4)_6H_2W_{12}O_{40}$/DMF/H_2O all show similar absorption patterns without significant peaks, implying that there are no direct strong intermolecular interactions between PEO-*b*-PS and $(NH_4)_6H_2W_{12}O_{40}$. Furthermore, for comparison study, we prepared another assembly system by mixing P4VP-*b*-PS and acidic POMs ($H_3PW_{12}O_{40}$) in DMF/H_2O (9:1 v/v). This well-documented acidic POMs/polymer assembly system by virtue of direct intermolecular interactions exhibits a totally different UV-vis pattern (**Supplementary Fig. 7**b), compared to our proposed non-acidic POMs/PEO-*b*-PS assembly system, namely the SPEA process (Supplementary Fig. 7a). The UV-vis analysis reveals that, compared with P4VP-*b*-PS/DMF/H_2O and $H_3PW_{12}O_{40}$/DMF/H_2O, the P4VP-*b*-PS/$H_3PW_{12}O_{40}$/DMF/H_2O has a new absorption peak at 500 nm, due to the formation of protonated pyridine group enabling the strong electrostatic interactions between positively charged P4VP segments and $PW_{12}O_{40}^{3-}$ anions (inset in Supplementary Fig. 7b).

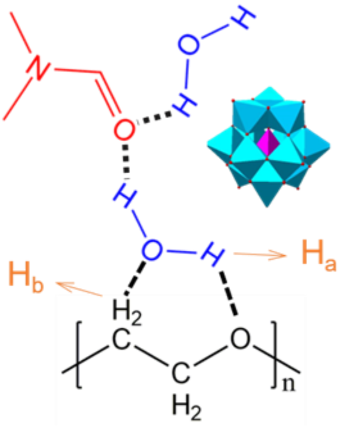

Supplementary Fig. 8. Schematic illustration of the underlying intermolecular interactions among PEO, non-acidic POMs and DMF·nH_2O complexes for the SPEA process.

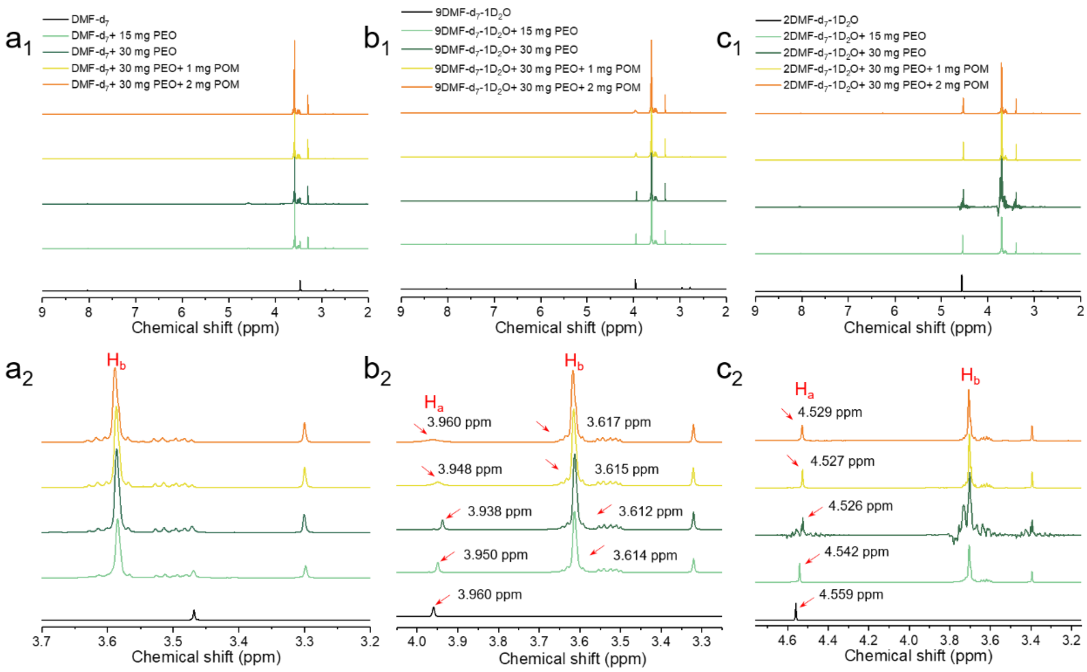

Supplementary Fig. 9. ^{1}H NMR spectra of PEO/POMs mixture recorded in DMF-d_7 (a, inevitably containing trace amounts of H_2O due to the hygroscopicity of DMF), DMF-d_7/D_2O (b, 9:1 v/v) and DMF-d_7/D_2O (c, 2:1 v/v) solution. The amount of solvent is fixed at 0.5 ml.

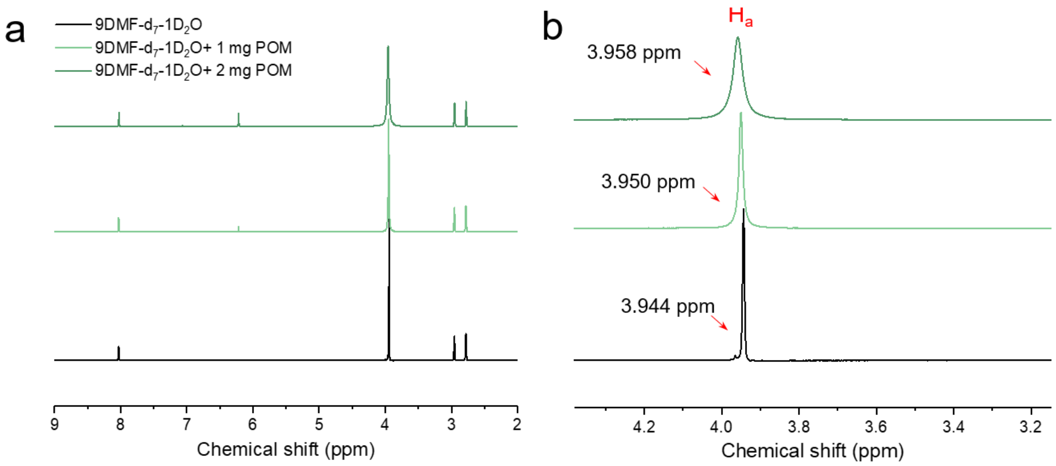

Supplementary Fig. 10. ^{1}H NMR spectra of POMs recorded in the range of 2-9 ppm (a) and 3.15-4.38 ppm (b) in DMF-d_7/D_2O (9:1 v/v) solution. The amount of solvent is 0.5 ml.

Further characterization was performed using ^{1}H NMR spectroscopy to investigate the interactions between the components. PEO (M_w = 350) homopolymer was used to replace the PEO-*b*-PS during the NMR characterization because the PEO segments were considered to interact with water molecules in the SPEA process and the long PS segments can suppress the signals of the components of interest, including PEO and H_2O.

A representative result of SPEA process in DMF-d_7/D_2O (9:1 v/v) is presented in **Supplementary Fig. 9**. As the content of PEO increases, the H_a (hydrogen of H_2O) signal shifts upfield. Meanwhile, the H_b (hydrogen of methylene in PEO) signal also shifts upfield but the amplitude is smaller. These results indicate that PEO interacts with H_2O through double hydrogen bonding in opposite directions. Into this system, we further introduce POMs ($(NH_4)_6H_2W_{12}O_{40}$), which can induce the signal of H_a to shift downfield (**Supplementary Fig. 9b**). For comparison, we directly add POMs to DMF-d_7/D_2O (9:1 v/v) and we found that as the content of POMs increases, the H_a signal also shifts downfield (**Supplementary Fig. 10**) but the amplitude is smaller than that in Supplementary Fig. 9b. Therefore, the obvious chemical shifts of H_a in Supplementary Fig. 9b can be attributed to the following two aspects. Firstly, due to the oxygen-rich surface, POMs can interact with H_2O through hydrogen bonding (O−H···O). Secondly, the POMs-H_2O interaction can weaken the hydrogen bonding between PEO and H_2O. In other words, POMs and PEO simultaneously interact with

H_2O (or DMF·nH_2O complexes), which implies that H_2O can behave as a medium to connect with POMs and PEO. Moreover, the noticeable broadening of the H_a signal with the POMs content indicates that POMs have relatively strong interactions with H_2O, retaining H_2O molecules tightly around the POMs.

We further perform control experiments to validate the SPEA process. In pure DMF-d_7, the addition of PEO and POMs does not cause any significant chemical shift, indicating that there is no obvious interaction between the components in this system (Supplementary Fig. 9a). When the excessive content of H_2O is applied (Supplementary Fig. 9c, V_{DMF-d7}/V_{D2O} = 2:1, V_{D2O} ~ 33%), the addition of POMs only causes the H_a signal to shift downfield slightly, and this shift amplitude is much smaller than that in the 9DMF-d_7-1D_2O system, and no obvious broadening occurs. It suggests that the interactions between POMs and H_2O are weak or only a small amount of H_2O can form hydrogen bonding with POMs. These findings based on ^{1}H NMR characterization are in good agreement with the explanation that most of the water molecules exist as H_2O clusters rather than DMF·nH_2O complexes in cases of high H_2O content (V_{H2O} > 20%). These results explain why the assembly of ordered structures cannot be achieved in systems with high H_2O content, that is, only water molecules which exist as DMF·nH_2O complexes can precisely allocate POMs at the PEO region.

5. Co-assembly of PEO-*b*-PS micelles and POMs

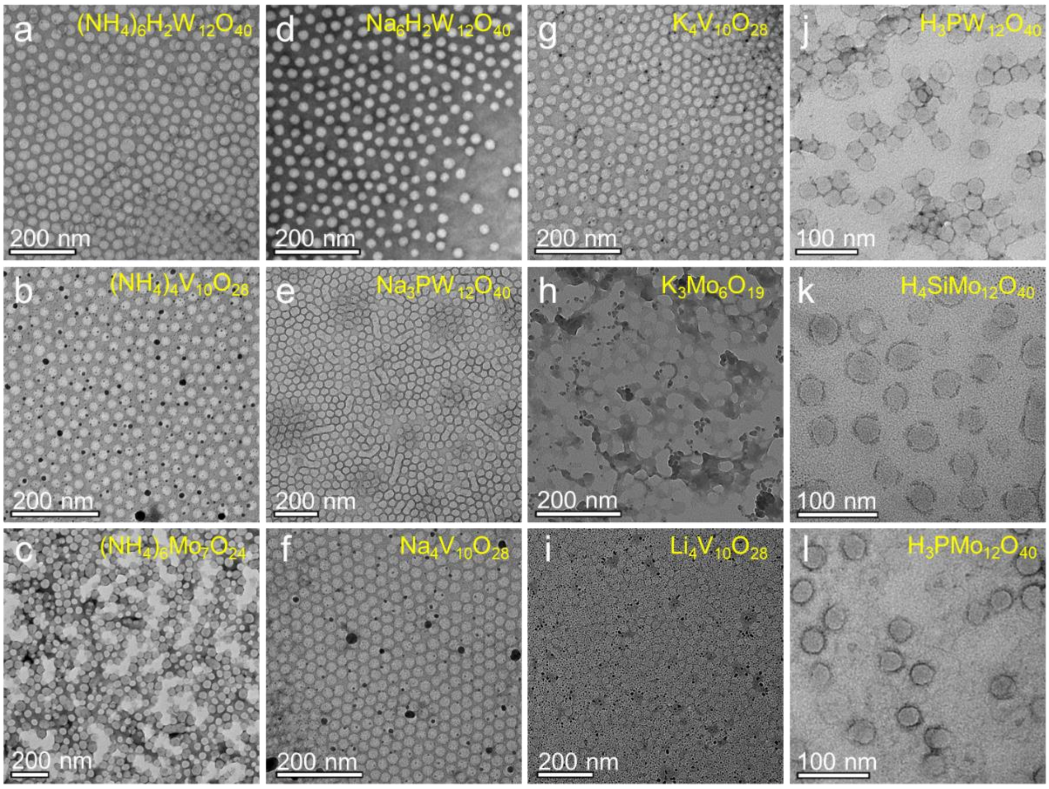

Supplementary Fig. 11. (a-l) TEM images of the co-assembly between PEO-*b*-PS micelles with $(NH_4)_6H_2W_{12}O_{40}$ (a), $(NH_4)_4V_{10}O_{28}$ (b), $(NH_4)_6Mo_7O_{24}$ (c), $Na_6H_2W_{12}O_{40}$ (d), $Na_3PW_{12}O_{40}$ (e), $Na_4V_{10}O_{28}$ (f), $K_4V_{10}O_{28}$ (g), $K_3Mo_6O_{19}$ (h), $Li_4V_{10}O_{28}$ (i), $H_3PW_{12}O_{40}$ (j), $H_4SiMo_{12}O_{40}$ (k) and $H_3PMo_{12}O_{40}$ (l), respectively. The samples were prepared by directly dropping as-prepared diluted PEO-*b*-PS/POMs co-assembly system onto carbon-coated copper grids.

Supplementary Fig. 11 shows the high-contrast transmission electron microscopy (HCTEM) images for the as-prepared PEO-*b*-PS/POMs co-assembly on carbon-coated copper grids. In order to observe the interactions between PEO-*b*-PS monomicelles (PS as a core and PEO as a shell) and POMs, the PEO-*b*-PS/POMs co-assembly system was diluted with fivefold DMF and H_2O to avoid the close packing of micelles.

Similar to the heteropolyacid staining process, the bright region is PS segment and the dark region is POMs due to the high mass contrast of POMs, while the

location of PEO segment cannot be precisely defined. For 9 kinds of non-acidic POMs assembly, the bright micelles were embedded in uniform dark POM matrix and no obvious aggregation of POMs in the edge of micelles was observed, indicating the relatively weak interactions between non-acidic POMs and micelles (Supplementary Fig. 11a-i). By contrast, in the case of acidic POMs (i.e., heteropolyacid), the boundary of PEO-*b*-PS micelles can be easily visible due to the strong Coulombic attraction between POM anions and protonated PEO shell (Supplementary Fig. 11j-l).

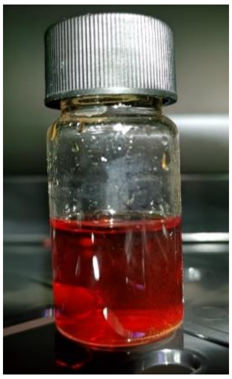

Supplementary Fig. 12. Optical photograph of $(NH_4)_4V_{10}O_{28}$ and PEO-*b*-PS dissolved in DMF/H_2O

For the coassembly of vanadium-based POM ($(NH_4)_4V_{10}O_{28}$, $Na_4V_{10}O_{28}$, $K_4V_{10}O_{28}$ and $Li_4V_{10}O_{28}$) and PEO-*b*-PS, molecular vanadate salts (NH_4VO_3, Na_3VO_4, KVO_3 and $LiVO_3$, see Figure S15 for their XRD patterns) were used as precursors, which are insoluble in both DMF and H_2O. Trace amounts of hydrochloric acid aqueous solution was added to tune the pH value of the system. As the concentration of H^+ increases, the oxygen in vanadate is gradually replaced by H^+ and the ratio of oxygen to vanadium decreases successively, forming water-soluble vanadium-based POMs. The process is as follows: VO_3^- (colorless)-$V_2O_7^{4-}$ (colorless)-$V_3O_9^{4-}$ (colorless)-$V_{10}O_{28}^{4-}$ (orange)-V_2O_5 (red)-VO_2^+ (light yellow). Through adjusting the

concentration of hydrochloric acid (0.02-0.05 mol/L), $V_{10}O_{28}^{4-}$ can be obtained and homogeneously dissolved in DMF/H_2O binary solvent, forming transparent orange colloidal solution (**Supplementary Fig. 12**), which can be subsequently used to assemble with PEO-*b*-PS micelles. Due to the presence of Cl^- after introduction of HCl solution, small amounts of chlorate particles (NH_4Cl, NaCl, KCl, LiCl) can form and precipitate with the evaporation of solvent (Supplementary Fig. 11b, f-i). These trace amounts of salt can be washed away with H_2O in the final step of the synthesis. Similarly, $K_3Mo_6O_{19}$ is also suitable in this assembly system by using K_2MoO_4 as a precursor.

6. TG and XRD analysis

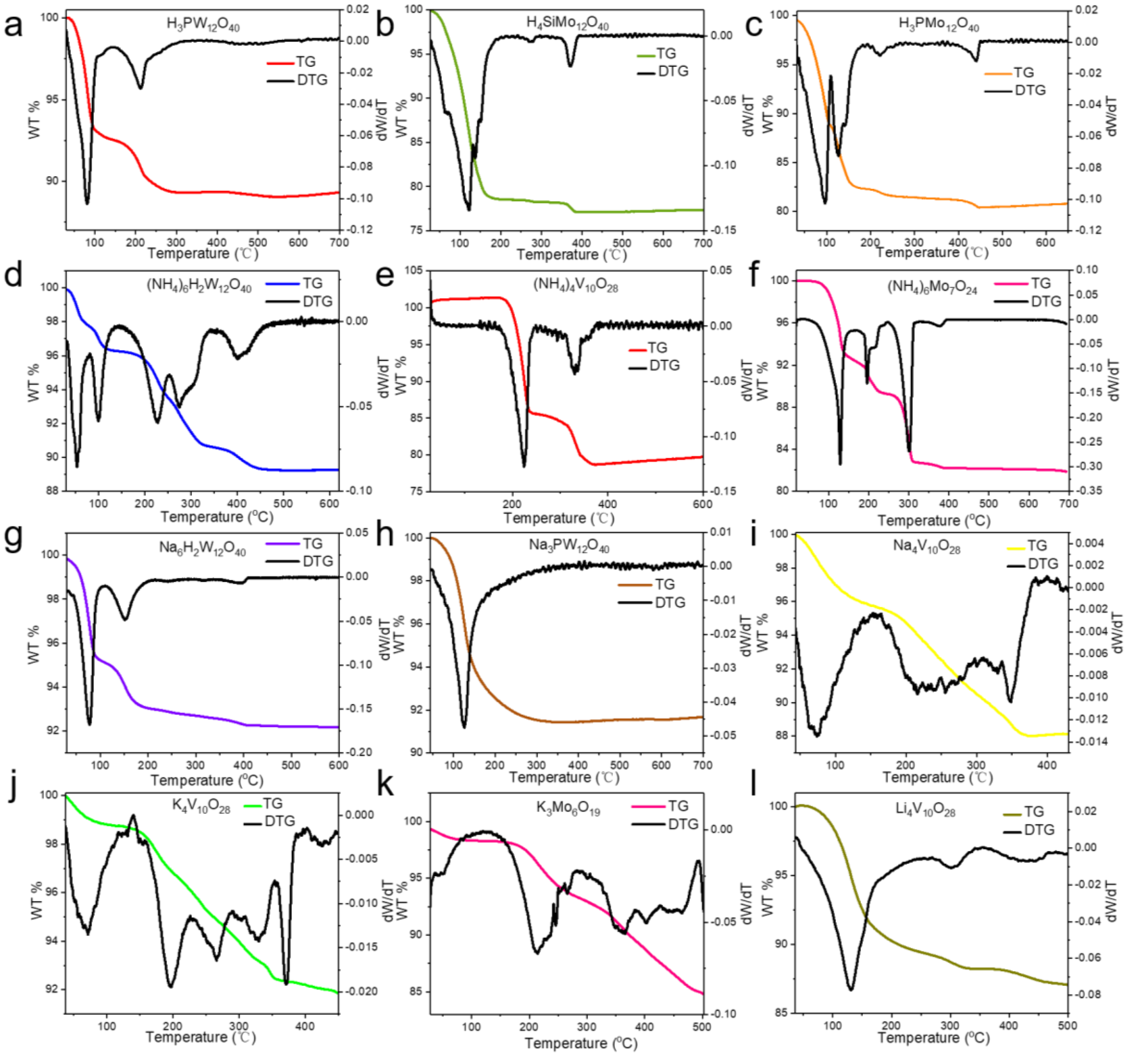

Supplementary Fig. 13. (a-l) TG and DTG curves of $H_3PW_{12}O_{40}$ (a), $H_4SiMo_{12}O_{40}$ (b), $H_3PMo_{12}O_{40}$ (c), $(NH_4)_6H_2W_{12}O_{40}$ (d), $(NH_4)_4V_{10}O_{28}$ (e), $(NH_4)_6Mo_7O_{24}$ (f), $Na_6H_2W_{12}O_{40}$ (g), $Na_3PW_{12}O_{40}$ (h), $Na_4V_{10}O_{28}$ (i), $K_4V_{10}O_{28}$ (j), $K_3Mo_6O_{19}$ (k) and $Li_4V_{10}O_{28}$ (l).

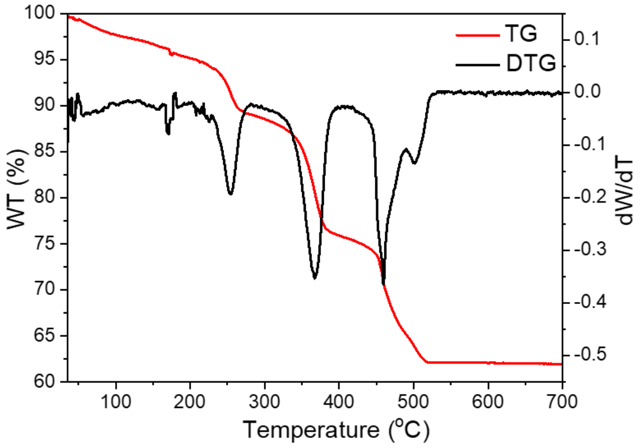

Supplementary Fig. 14. TG and DTG curves of PEO-*b*-PS/$(NH_4)_6H_2W_{12}O_{40}$ composite.

Thermogravimetry (TG) and differential thermogravimetry curves (DTG) were recorded to identify the optimal thermal decomposition temperature of POMs into metal oxides. Treatment at 400 °C was performed to decompose $(NH_4)_6Mo_7O_{24}$, $H_4SiMo_{12}O_{40}$, $(NH_4)_4V_{10}O_{28}$, $Na_4V_{10}O_{28}$, $K_4V_{10}O_{28}$, $Li_4V_{10}O_{28}$ and $K_3Mo_{10}O_{29}$. Treatment at 450 °C was performed to decompose $Na_3PW_{12}O_{40}$ and $H_3PMo_{12}O_{40}$. Treatment at 500 °C was performed to decompose $(NH_4)_6H_2W_{12}O_{40}$ and $Na_6H_2W_{12}O_{40}$. Treatment at 550 °C was performed to decompose $H_3PW_{12}O_{40}$. The decomposition temperature of PEO-b-PS/$(NH_4)_6H_2W_{12}O_{40}$ is close to $(NH_4)_6H_2W_{12}O_{40}$, indicating that the existence of copolymers has no significant effect on the decomposition of POMs.

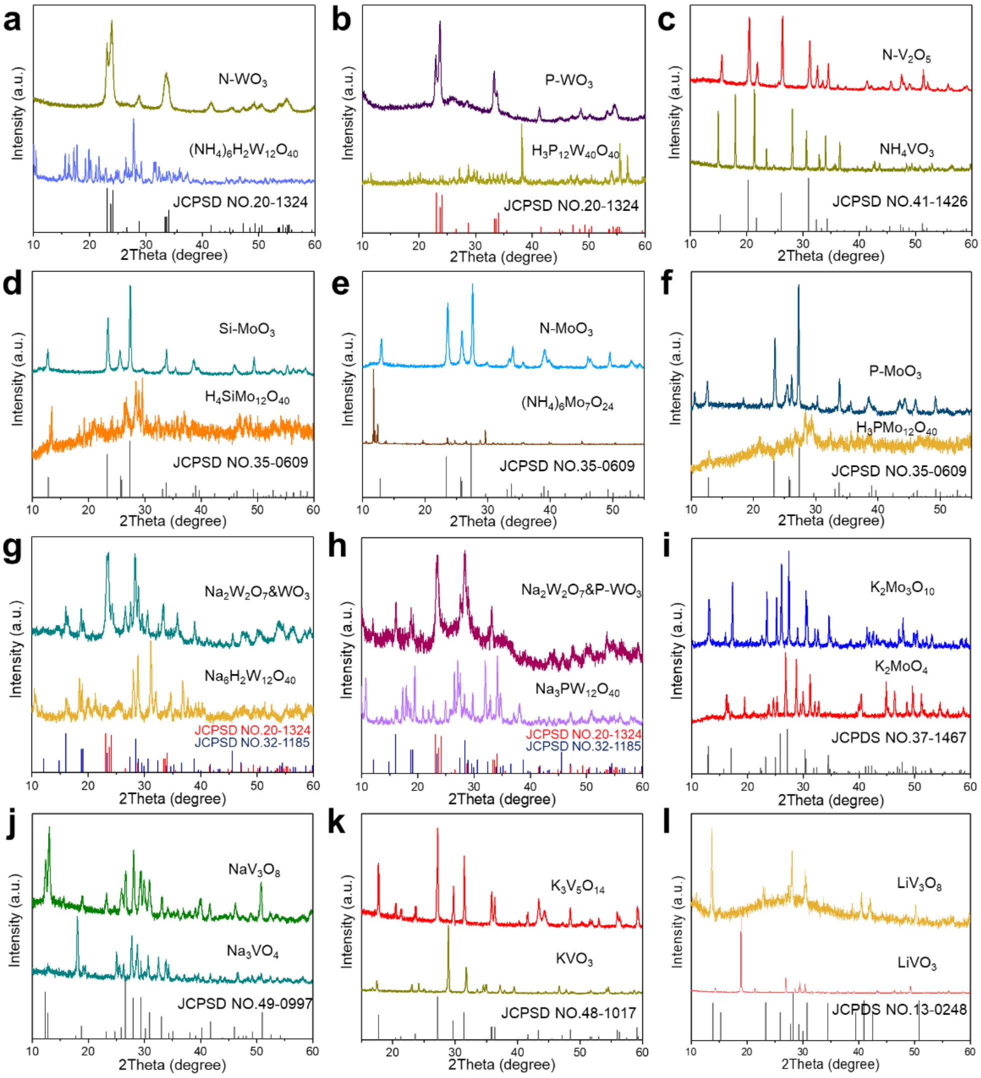

Supplementary Fig. 15. (a-l) XRD patterns of mesoporous N-WO_3 (a), P-WO_3 (b), N-V_2O_5 (c), Si-MoO_3 (d), N-MoO_3 (e), P-MoO_3 (f), $Na_2W_2O_7$&WO_3 (g), $Na_2W_2O_7$&P-WO_3 (h), $K_2Mo_3O_{10}$ (i), NaV_3O_8 (j), $K_3V_5O_{14}$ (k), LiV_3O_8 (j) and corresponding POMs or molecular precursors, respectively.

To verify the decomposition of POMs and identify the crystalline phases of the obtained metal oxides, we further carried out the XRD measurements. $(NH_4)_4V_{10}O_{28}$, $Na_4V_{10}O_{28}$, $K_4V_{10}O_{28}$, $Li_4V_{10}O_{28}$ and $K_3Mo_{10}O_{29}$ were prepared following the method in **supplementary section 5**. Other POMs were purchased and used as received.

7. TEM characterization of $mK_2Mo_3O_{10}$ and $mLiV_3O_8$

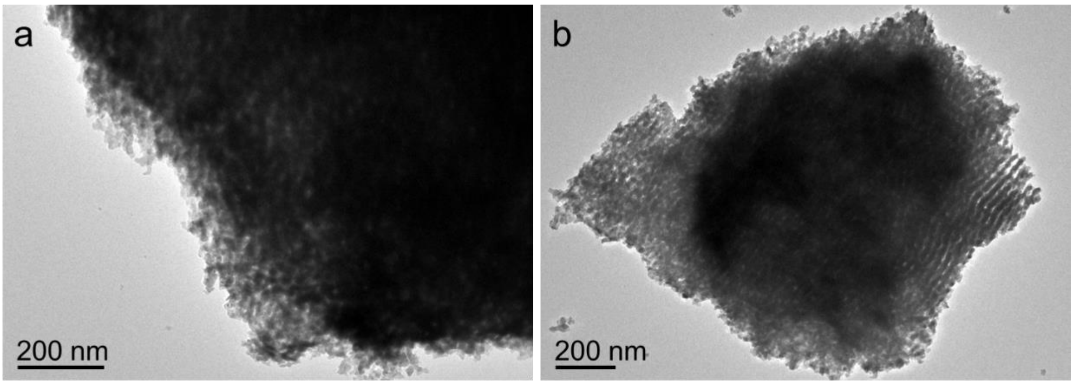

Supplementary Fig. 16. TEM images of $mK_2Mo_3O_{10}$ (a) and $mLiV_3O_8$ (b). Mesoporous $K_2Mo_3O_{10}$ and LiV_3O_8 materials possess short-range order and wormlike mesopores.

8. XPS and EDX element mapping of mMOs

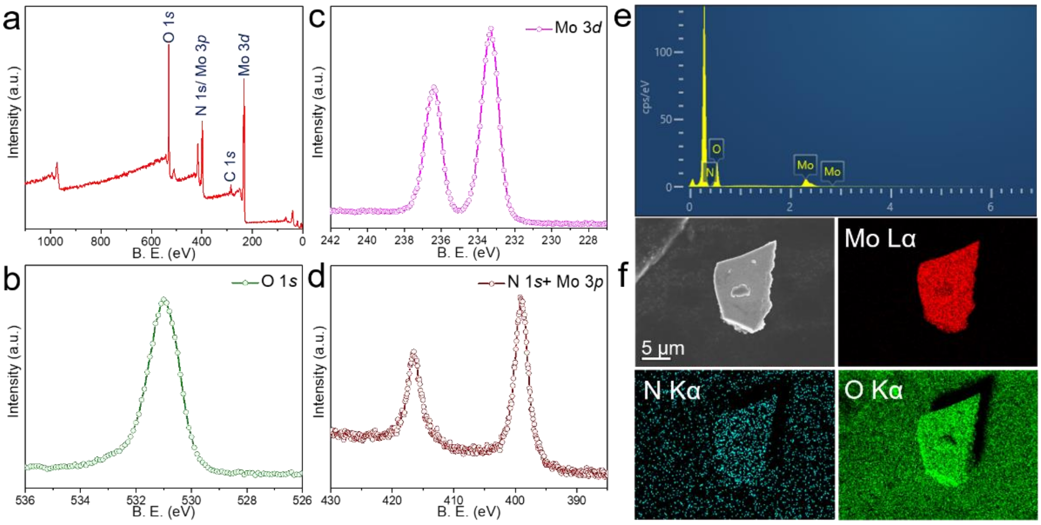

Supplementary Fig. 17. X-ray photoelectron spectra (XPS) of the samples (a) full spectrum, (b) O 1*s*, (c) Mo 3*d* and (d) N 1*s*+ Mo 3*p* core level peak regions of the mN-MoO_3 prepared with $(NH_4)_6Mo_7O_{24}$. (e) Energy dispersive spectrum of mN-MoO_3. (f) Element mapping shows the uniform distribution of Mo, N and O throughout the mN-MoO_3.

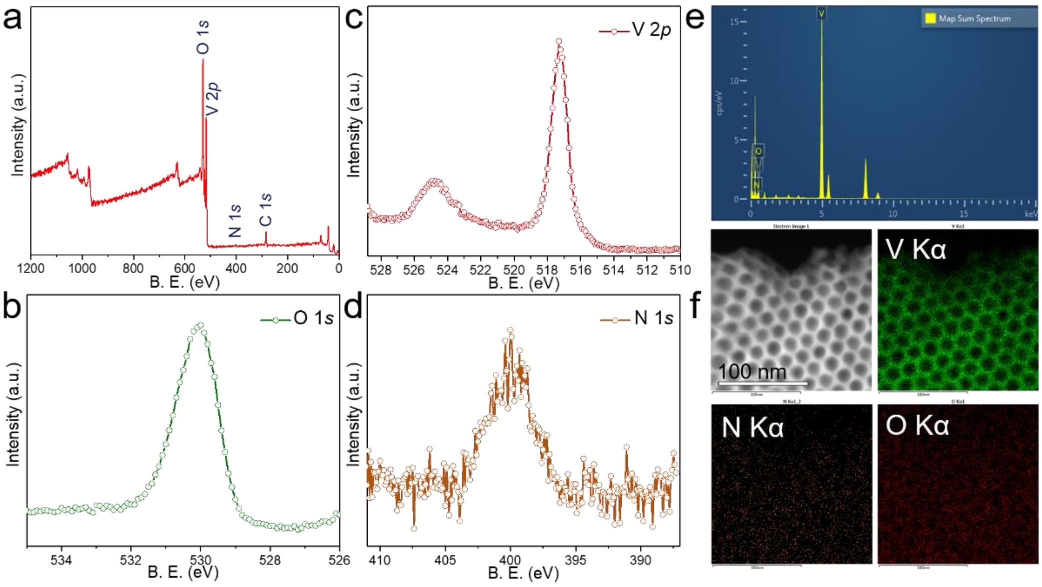

Supplementary Fig. 18. XPS of the samples (a) full spectrum, (b) O 1*s*, (c) V 2*p* and (d) N 1*s* core level peak regions of the mN-V_2O_5 prepared with $(NH_4)_4V_{10}O_{28}$. (e) Energy dispersive spectrum of mN-V_2O_5. (f) Element mapping shows the uniform distribution of V, N and O throughout the mN-V_2O_5.

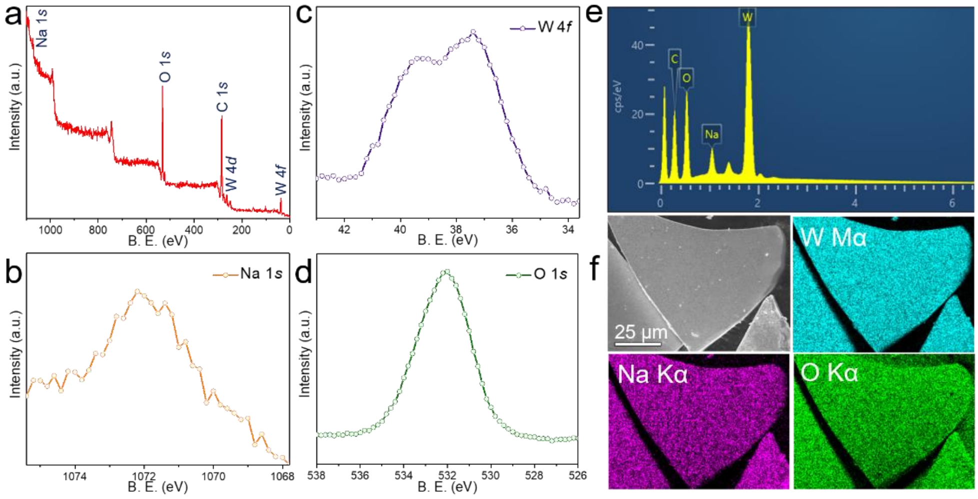

Supplementary Fig. 19. XPS of the samples (a) full spectrum, (b) Na 1*s*, (c) W 4*f* and (d) O 1*s* core level peak regions of the m$Na_2W_2O_7$&WO_3 prepared with $Na_6H_2W_{12}O_{40}$. (e) Energy dispersive spectrum of m$Na_2W_2O_7$&WO_3. (f) Element mapping shows the uniform distribution of W, Na and O throughout the m$Na_2W_2O_7$&WO_3.

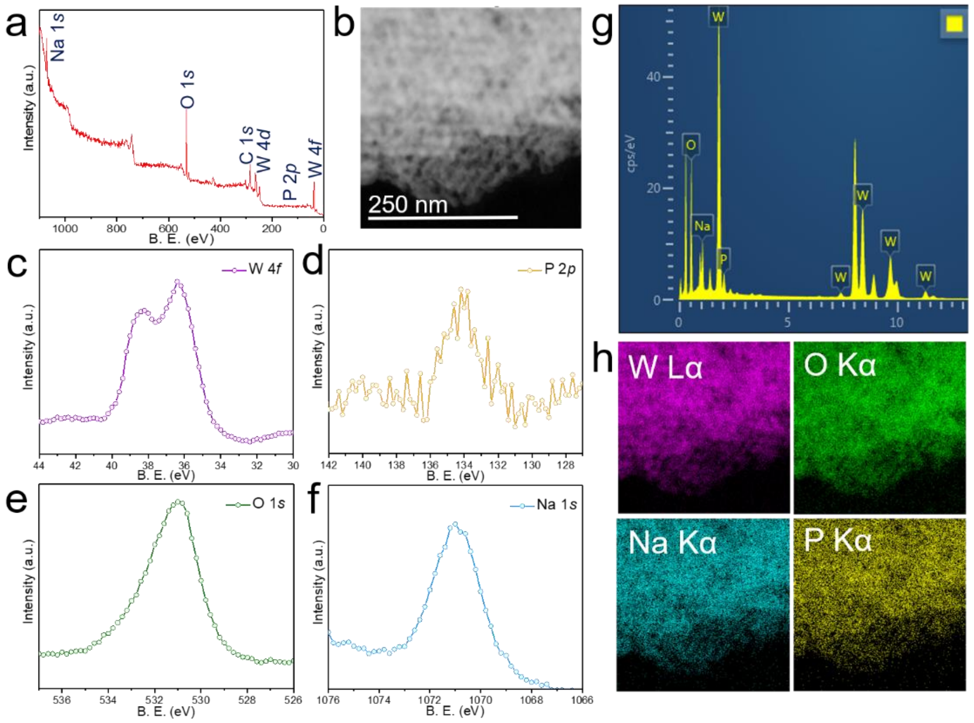

Supplementary Fig. 20. XPS of the samples (a) full spectrum, (c) W 4*f*, (d) P 2*p*, (e) O 1*s* and (f) Na 1*s* core level peak regions of the mP-$Na_2W_2O_7$&WO_3 prepared with $Na_3PW_{12}O_{40}$. (g) Energy dispersive spectrum of mP-$Na_2W_2O_7$&WO_3. (b and h) Element mapping shows the uniform distribution of W, O, Na and P throughout the mP-$Na_2W_2O_7$&WO_3.

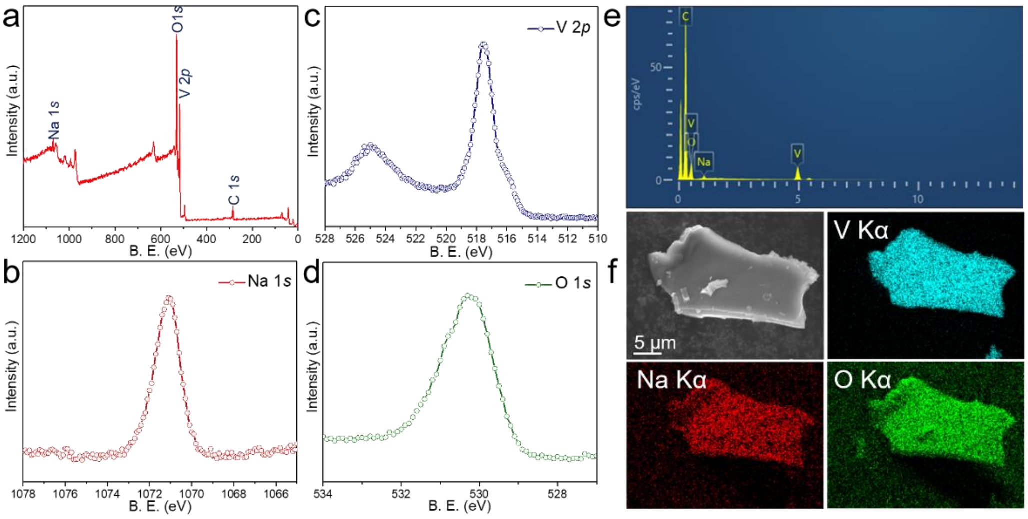

Supplementary Fig. 21. XPS of the samples (a) full spectrum, (b) Na 1*s*, (c) V 2*p* and (d) O 1*s* core level peak regions of the mNaV_3O_8 prepared with $Na_4V_{10}O_{28}$. (e) Energy dispersive spectrum of mNaV_3O_8. (f) Element mapping shows the uniform distribution of V, Na and O throughout the mNaV_3O_8.

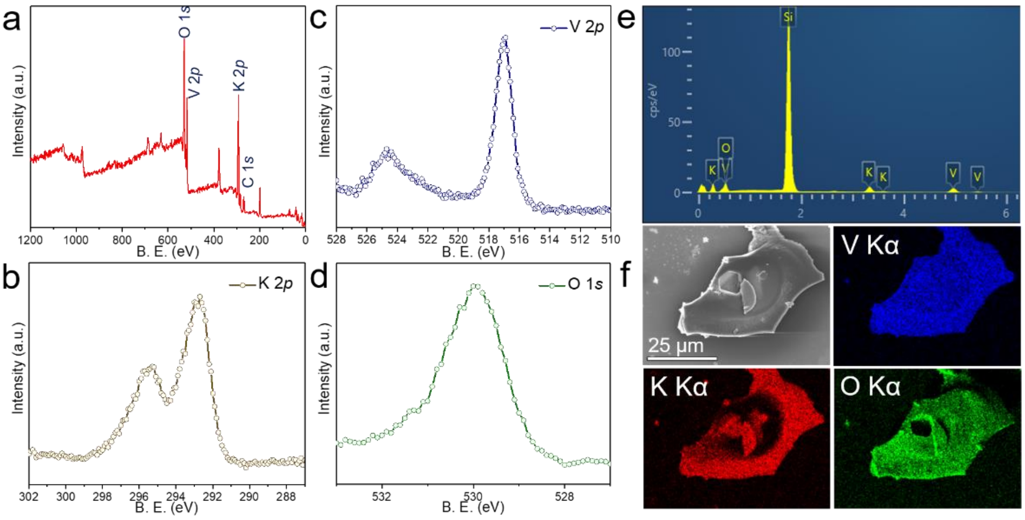

Supplementary Fig. 22. XPS of the samples (a) full spectrum, (b) K 2*p*, (c) V 2*p* and (d) O 1*s* core level peak regions of the m$K_3V_5O_{14}$ prepared with $K_4V_{10}O_{28}$. (e) Energy dispersive spectrum of m$K_3V_5O_{14}$. (f) Element mapping shows the uniform distribution of V, K and O throughout the m$K_3V_5O_{14}$.

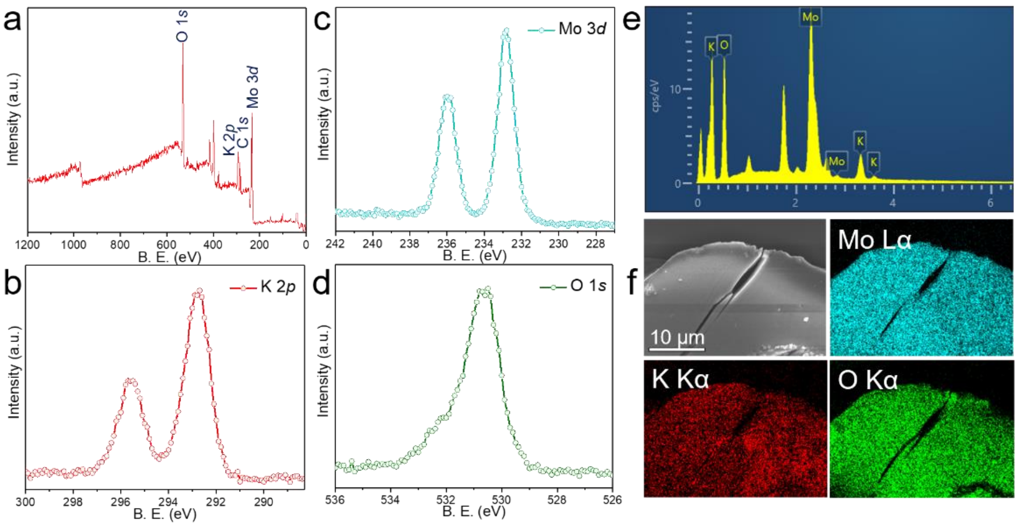

Supplementary Fig. 23. XPS of the samples (a) full spectrum, (b) K 2*p*, (c) Mo 3*d* and (d) O 1*s* core level peak regions of the m$K_2Mo_3O_{10}$ prepared with $K_3Mo_6O_{19}$. (e) Energy dispersive spectrum of m$K_2Mo_3O_{10}$. (f) Element mapping shows the uniform distribution of Mo, K and O throughout the m$K_2Mo_3O_{10}$.

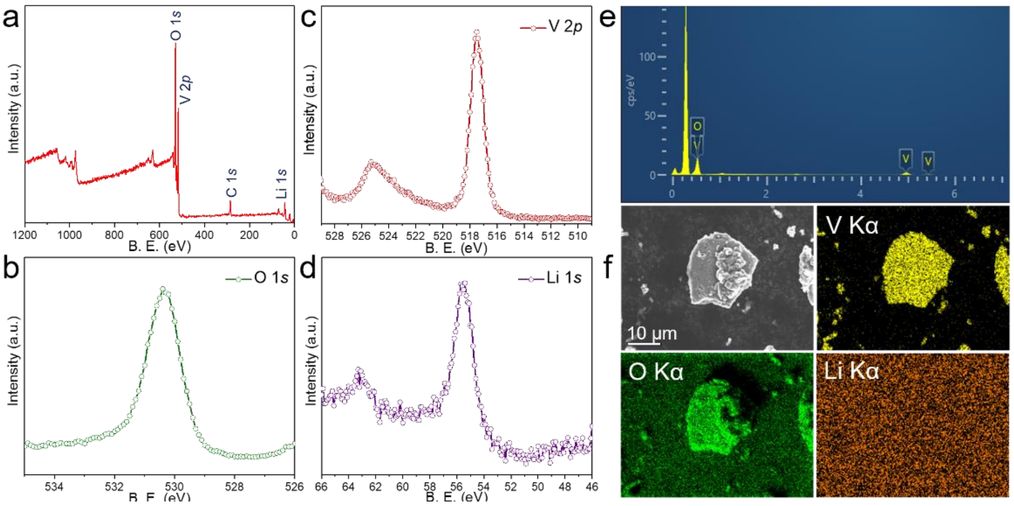

Supplementary Fig. 24. XPS of the samples (a) full spectrum, (b) O 1*s*, (c) V 2*p* and (d) Li 1*s* core level peak regions of the mLiV_3O_8 prepared with $Li_4V_{10}O_{28}$. (e) Energy dispersive spectrum of mLiV_3O_8. (f) Element mapping shows the uniform distribution of V and O throughout the mLiV_3O_8, while Li cannot be detected by EDS due to its low atomic number.

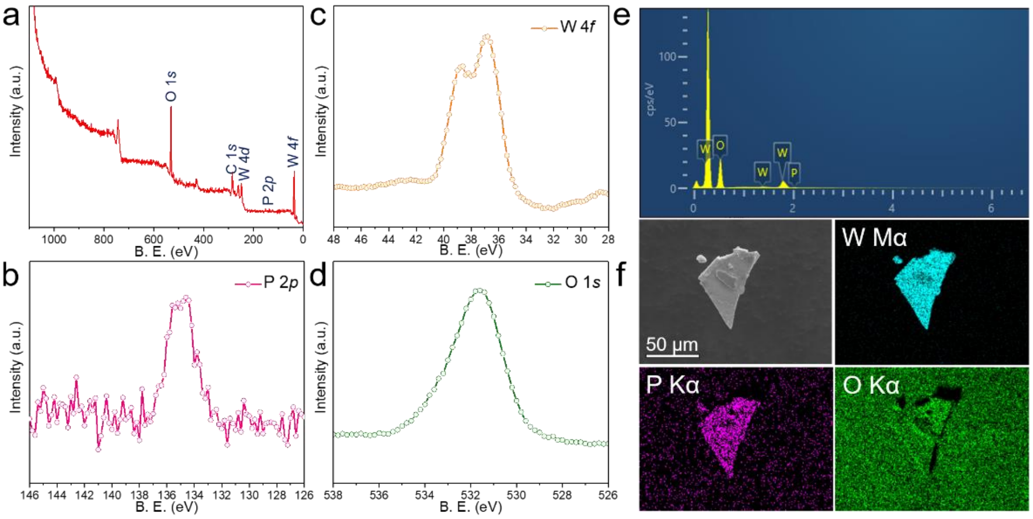

Supplementary Fig. 25. XPS of the samples (a) full spectrum, (b) P 2*p*, (c) W 4*f* and (d) O 1*s* core level peak regions of the mP-WO_3 prepared with $H_3PW_{12}O_{40}$. (e) Energy dispersive spectrum of mP-WO_3. (f) Element mapping shows the uniform distribution of W, P and O throughout the mP-WO_3.

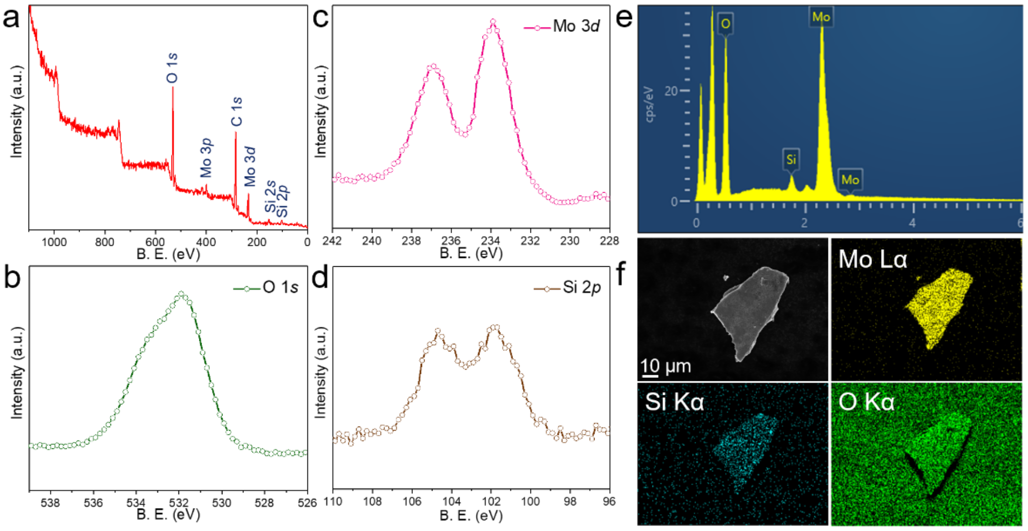

Supplementary Fig. 26. XPS of the samples (a) full spectrum, (b) O 1*s*, (c) Mo 3*d* and (d) Si 2*p* core level peak regions of the mSi-MoO_3 prepared with $H_4SiMo_{12}O_{40}$. (e) Energy dispersive spectrum of mSi-MoO_3. (f) Element mapping shows the uniform distribution of Mo, Si and O throughout the mSi-MoO_3.

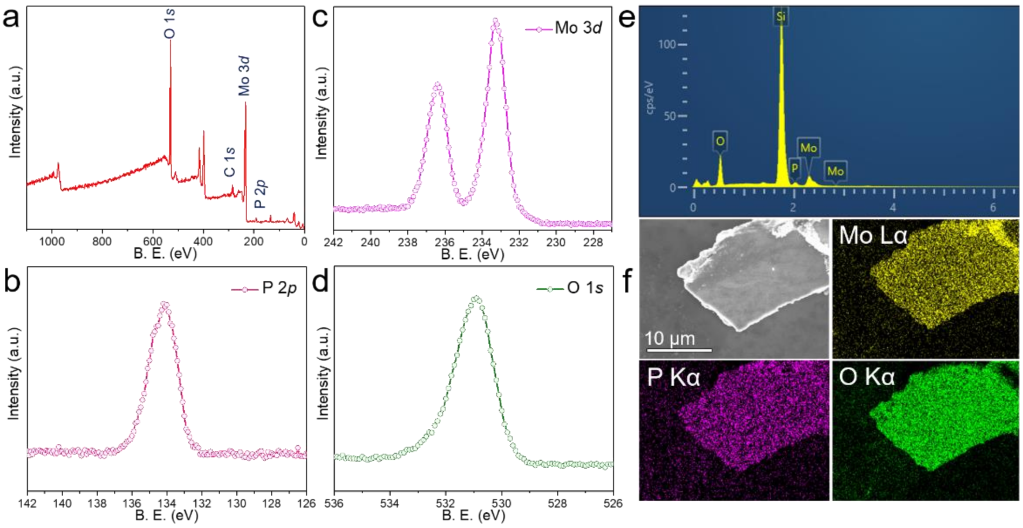

Supplementary Fig. 27. XPS of the samples (a) full spectrum, (b) P 2*p*, (c) Mo 3*d* and (d) O 1*s* core level peak regions of the mP-MoO_3 prepared with $H_3PMo_{12}O_{40}$. (e) Energy dispersive spectrum of mP-MoO_3. (f) Element mapping shows the uniform distribution of Mo, P and O throughout the mP-MoO_3.

9. SPEA enabled indirect co-assembly of POMs with other amphiphilic block copolymers

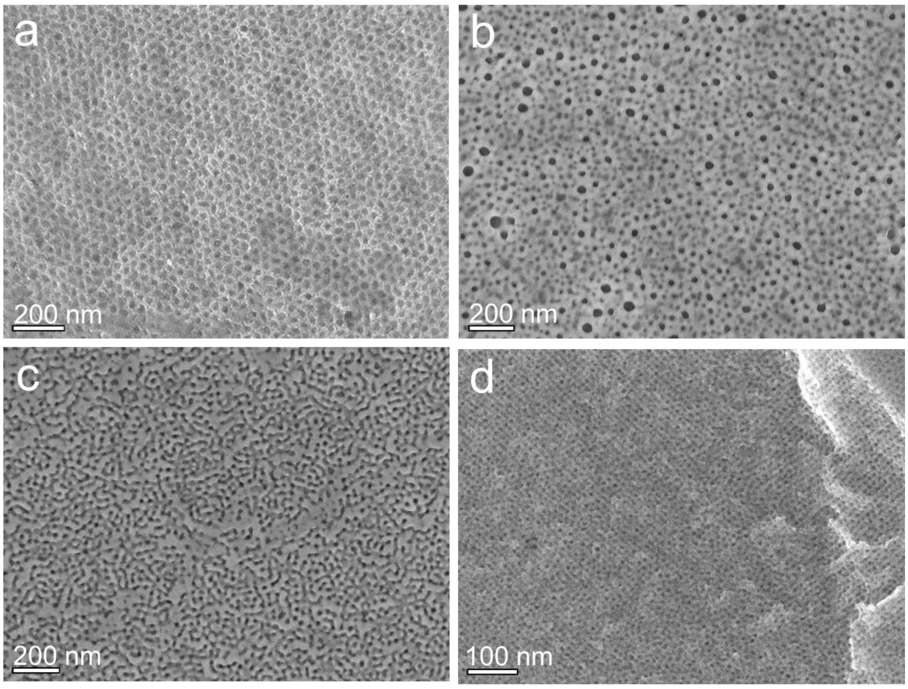

Supplementary Fig. 28. SEM images of mN-WO_3 synthesized by the co-assembly of AMT and different copolymers. (a) P4VP-*b*-PS, (b) PB-*b*-PEO, (c) PEO-*b*-PMMA and (d) PAA-*b*-PS.

Because SPEA method does not rely on the direct interaction between copolymers and inorganic precursors, it can be applicable for other linear amphiphilic diblock copolymers irrespective of the chemical properties of hydrophilic and hydrophobic segments. By using electroneutral poly(ethylene oxide)-*b*-poly(methyl methacrylate) (PEO-*b*-PMMA M_w = 17700 g/mol, polydispersity index (PDI) = 1.09), polybutylene-*b*-poly(ethyl oxide) (PB-*b*-PEO, M_w = 26500 g/mol, PDI = 1.20), positively charged poly(4-vinylpyridine)-*b*-polystyrene (P4VP-*b*-PS, M_w = 34200 g/mol, PDI = 1.18) and negatively charged poly(acrylic acid)-*b*-polystyrene (PAA-*b*-PS, M_w = 19600 g/mol, PDI = 1.12) as templates and AMT as precursor, mN-WO_3 can be successfully obtained.

10. Co-assembly parameters

10.1 Optimizing PEO-*b*-PS:AMT ratio

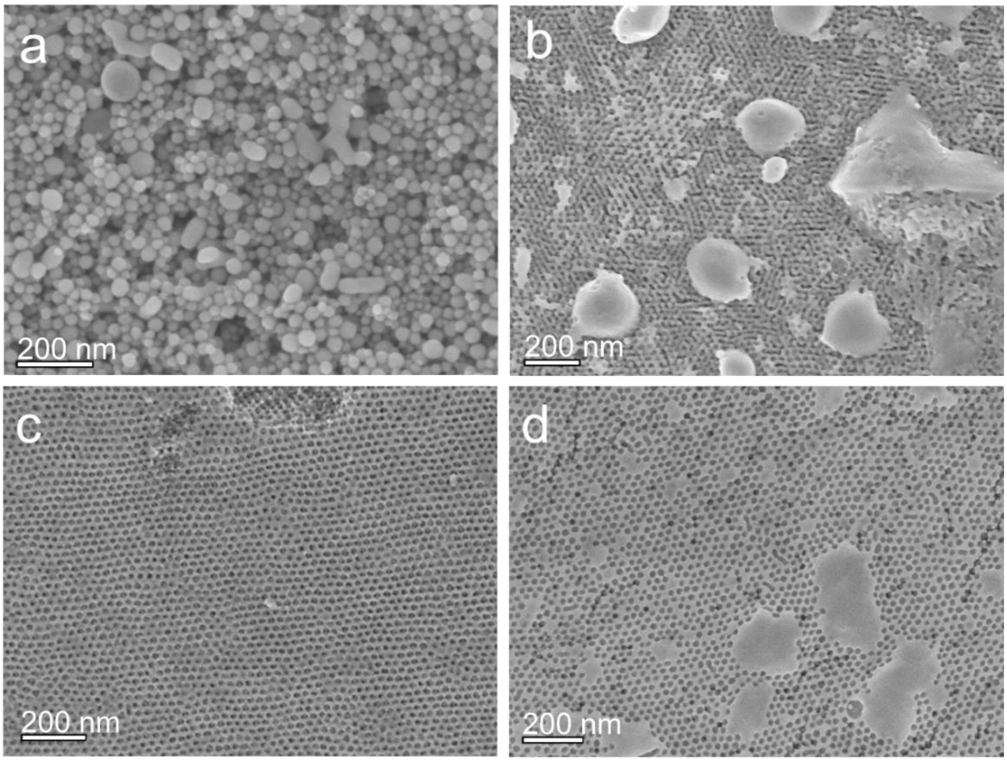

Supplementary Fig. 29. SEM images of mN-WO_3 synthesized using different mass ratios of PEO-*b*-PS to AMT. (a) 1:1, (b) 1:2, (c) 1:3 and (d) 1:4. The volume ratio of DMF to H_2O is fixed on 9:1.

The mass ratio of PEO-*b*-PS to AMT is finely tuned to form a highly ordered mesostructure. Insufficient AMT is unfavorable to support the framework, causing the collapse of the mesoporous structure after the thermal treatment, while excessive AMT can lead to undesired solid tungsten oxide particles deposited on the mesoporous matrix. Therefore, 1:3 was chosen as the optimum mass ratio of PEO-*b*-PS to AMT.

10.2 Optimizing DMF:H_2O ratio

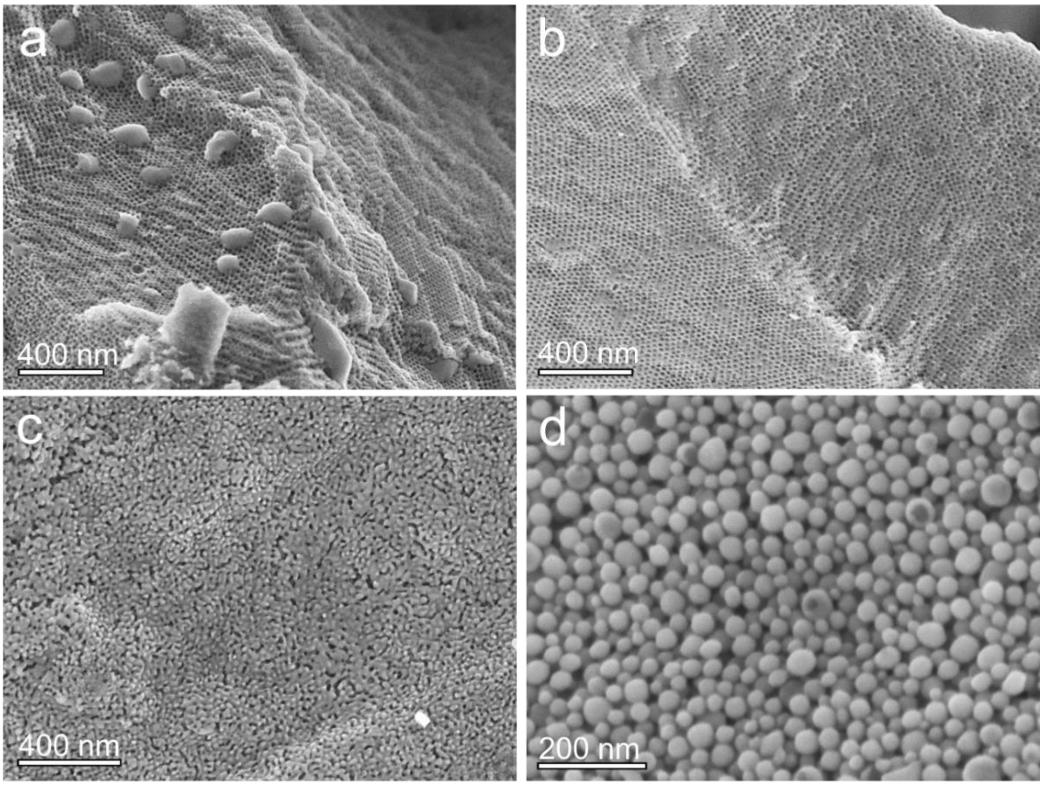

Supplementary Fig. 30. SEM images of mN-WO_3 synthesized using different volume ratios of DMF to H_2O. (a) 49:1, (b) 9:1, (c) 1:1 and (d) 1:4. The mass ratio of PEO-*b*-PS to AMT is fixed on 1:3.

10.3 Two-step sequential thermal treatment process

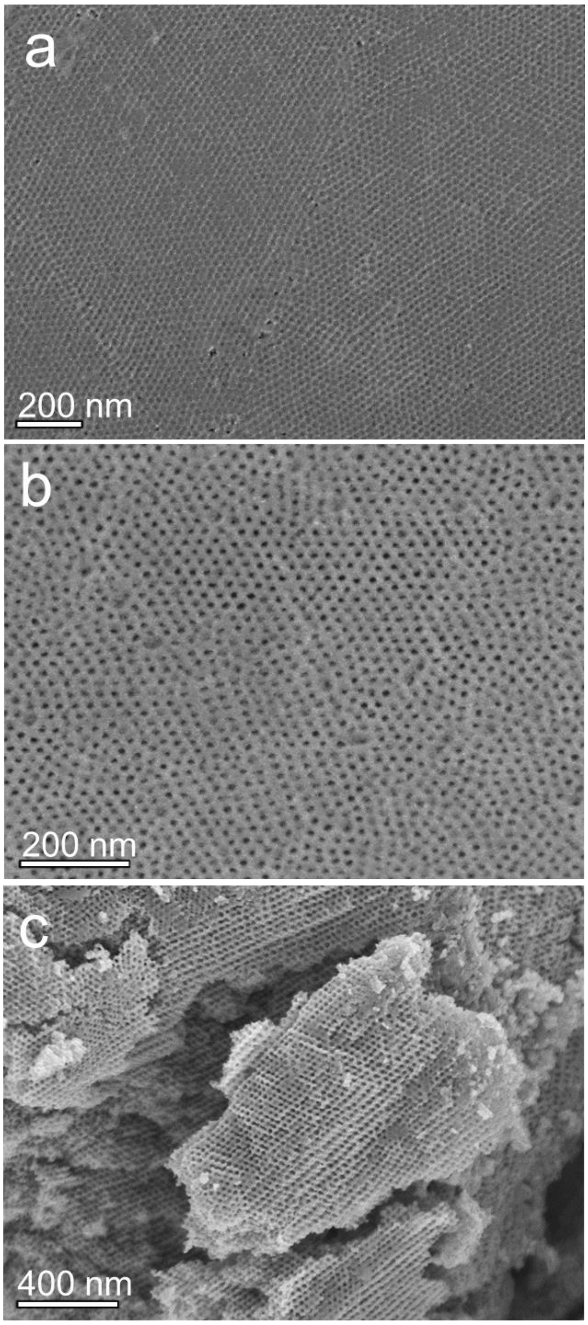

Supplementary Fig. 31. SEM images of the as-made PEO-*b*-PS/AMT composite powders before (a) and after (b) thermal treatment at 500 °C for 1 h in N_2 and then at 400 °C for 0.5 h in air (c).

11. Small angle X-ray scattering analysis

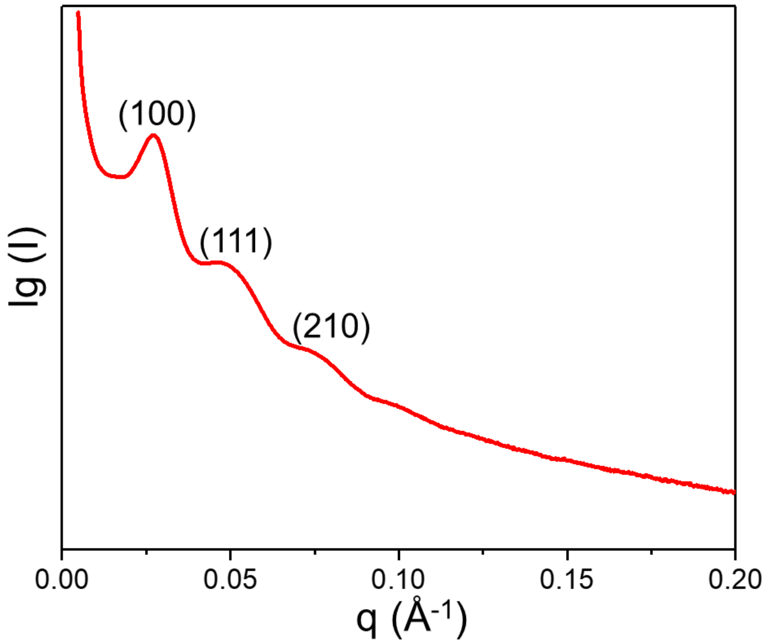

Supplementary Fig. 32. Small angle X-ray scattering spectrum of the mN-WO_3.

The spectrum shows three scattering peaks with *q*-values of 0.026, 0.047 and 0.077 Å^{-1}, corresponding to the (100), (111) and (210) reflections of ordered face-centered cubic mesostructured ($Fm\bar{3}m$), respectively. The *d*-spacing based on the strongest 100 peak was calculated to be 24.2 nm ($d_{100} = 2\pi/q$), which is consistent with the results of electron microscopy characterization (Figure 4).

12. Adjustment of pore sizes and pore structures

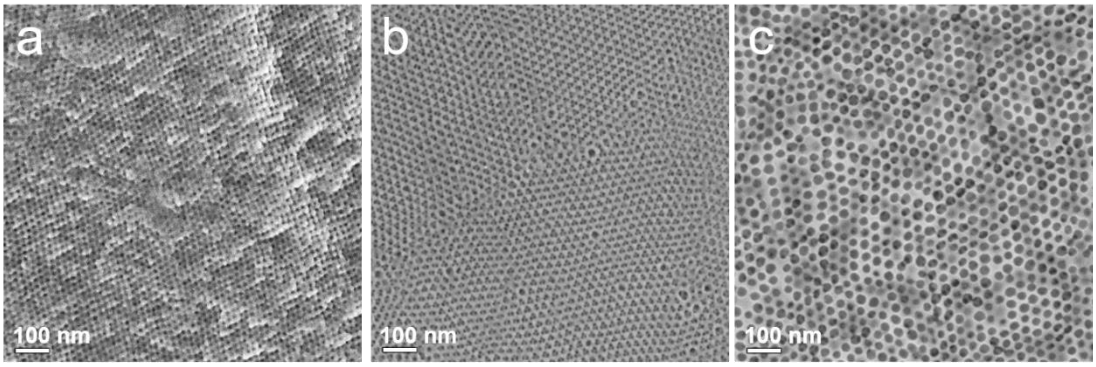

Supplementary Fig. 33. SEM images of mN-WO_3 synthesized by using PEO-*b*-PS with different molecular weights as templates. (a) PEO_{114}-*b*-PS_{103}, (b) PEO_{114}-*b*-PS_{175} and (c) PEO_{114}-*b*-PS_{250}.

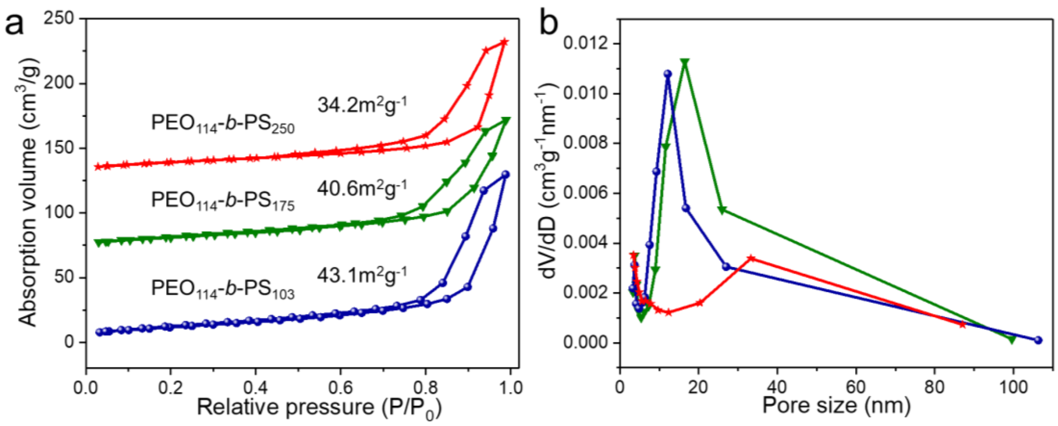

Supplementary Fig. 34. (a) The nitrogen adsorption-desorption isotherms (the samples prepared with PEO_{114}-*b*-PS_{175} and PEO_{114}-*b*-PS_{250} were offset vertically by 70 and 130 cm^3g^{-1}, respectively) and (b) corresponding pore-size distribution curves of the mN-WO_3 synthesized using PEO-*b*-PS with different molecular weight as templates: PEO_{114}-*b*-PS_{103} (blue curve), PEO_{114}-*b*-PS_{175} (green curve) and PEO_{114}-*b*-PS_{250} (red curve).

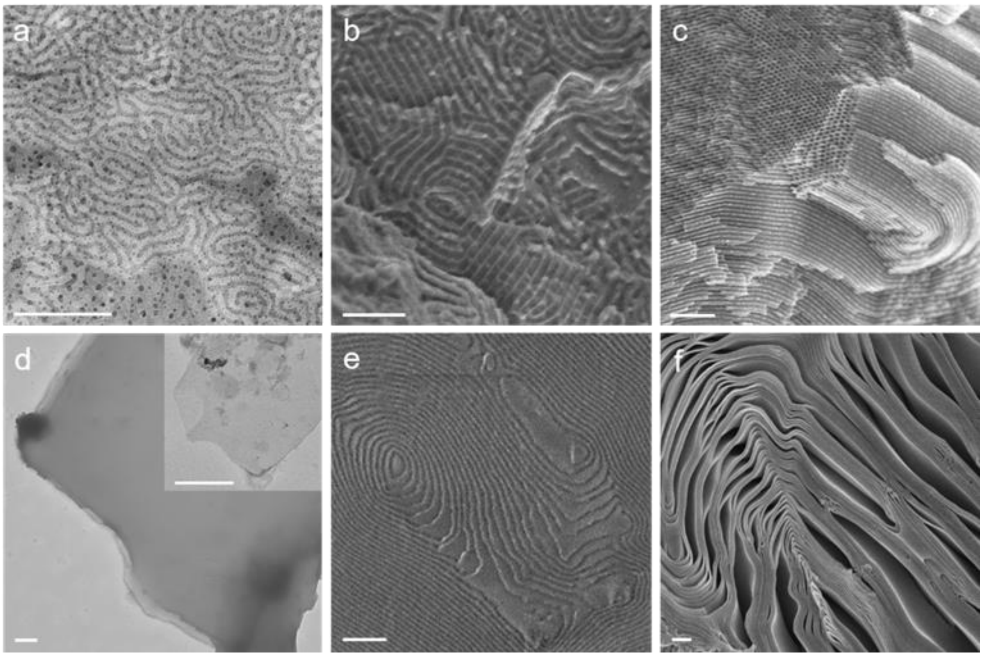

Supplementary Fig. 35. (a) TEM and (b) SEM images of as-made PEO_{114}-*b*-PS_{265}/AMT composites. (c) SEM image of mN-WO_3 synthesized using PEO_{114}-*b*-PS_{265} as template. (d and inset) TEM and (e) SEM images of as-made PEO_{114}-*b*-PS_{275}/AMT composites. (f) SEM image of mN-WO_3 synthesized using PEO_{114}-*b*-PS_{275} as template. The scale bars are 200 nm.

13. Electron microscopy characterization of mWO_3

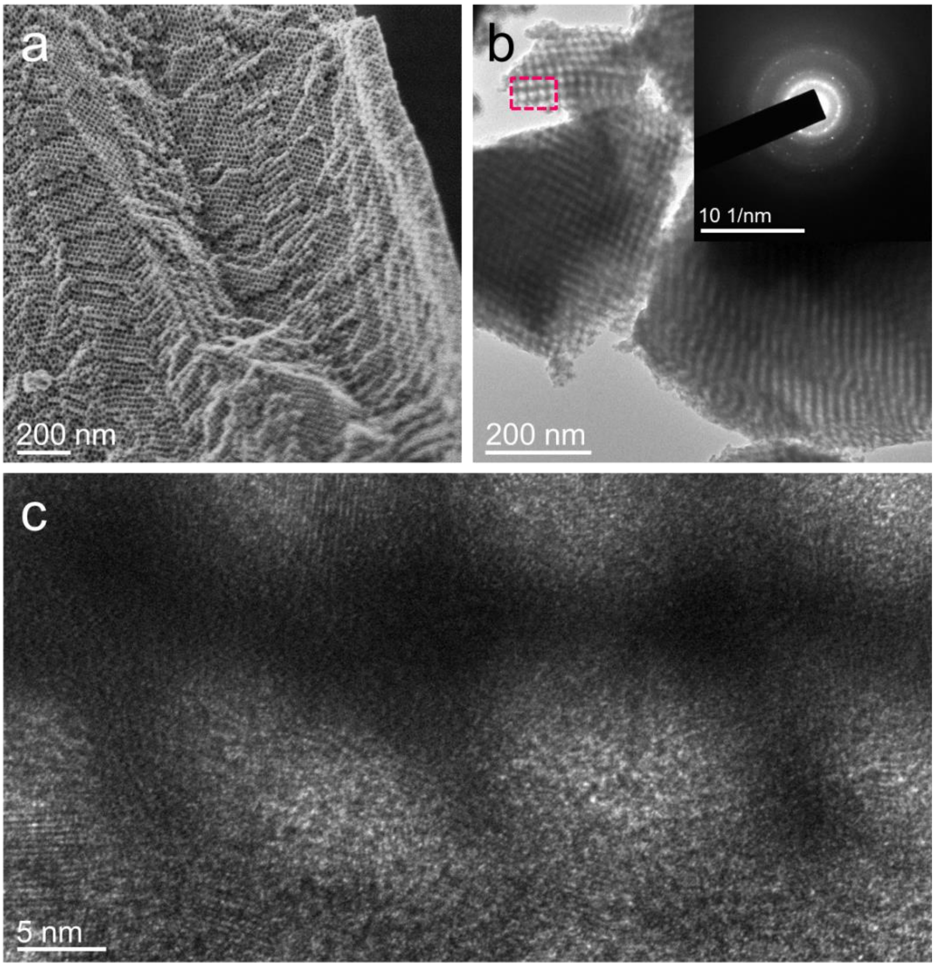

Supplementary Fig. 36. (a) Typical SEM and (b) TEM images with a selected-area electron diffraction pattern of mWO_3 by using WCl_6 as a precursor. (c) High-resolution TEM image of the selected area in (b). The sample was obtained after the thermal treatment at 500 °C in N_2 for 0.5 h and then at 400 °C for 0.5 h in air.

14. Thermal stability of mN-WO_3

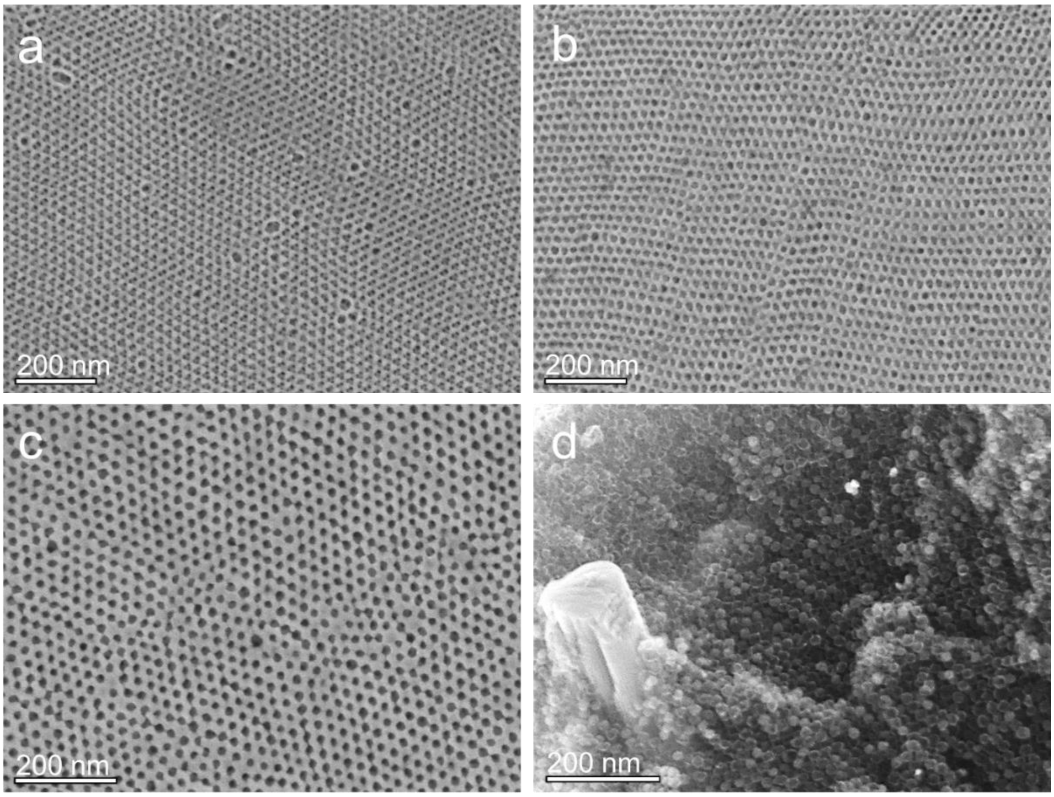

Supplementary Fig. 37. SEM images of mN-WO_3 synthesized by using AMT as tungsten precursor after the thermal treatment at 500 °C (a), 600 °C (b), 750 °C (c) and 800 °C (d) in N_2 for 0.5 h.

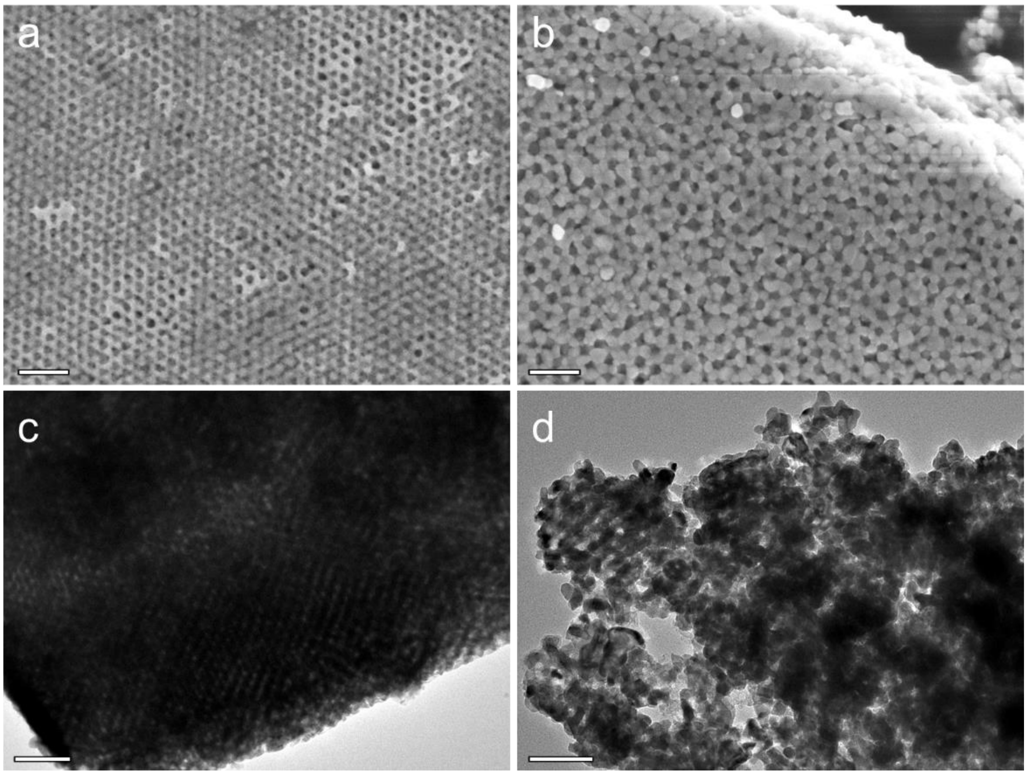

Supplementary Fig. 38. (a) SEM and (c) TEM images of mN-WO_3 synthesized by using AMT as tungsten precursor after the thermal treatment at 700 °C in N_2 for 0.5 h. (b) SEM and (d) TEM images of mWO_3 synthesized by using WCl_6 as a tungsten precursor after the thermal treatment at 700 °C in N_2 for 0.5 h. The scale bar is 100 nm.

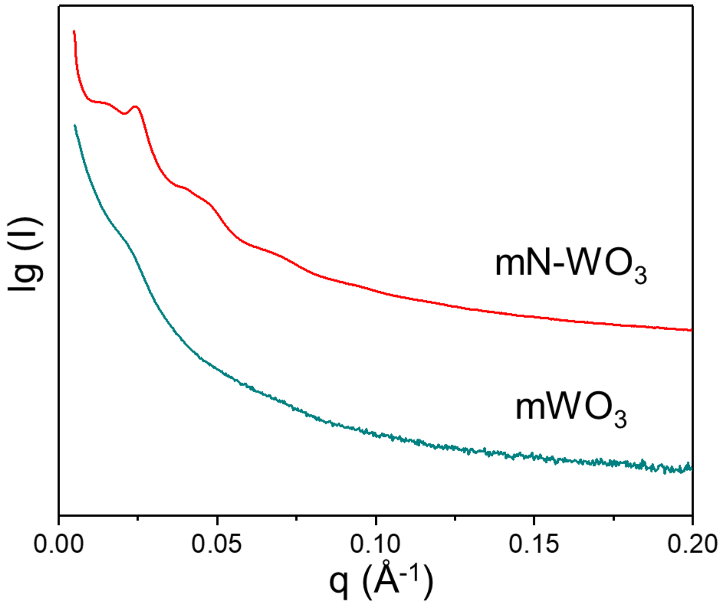

Supplementary Fig. 39. Small angle X-ray scattering spectra of the mN-WO_3 and mWO_3 after the thermal treatment at 700 °C in N_2 for 0.5 h.

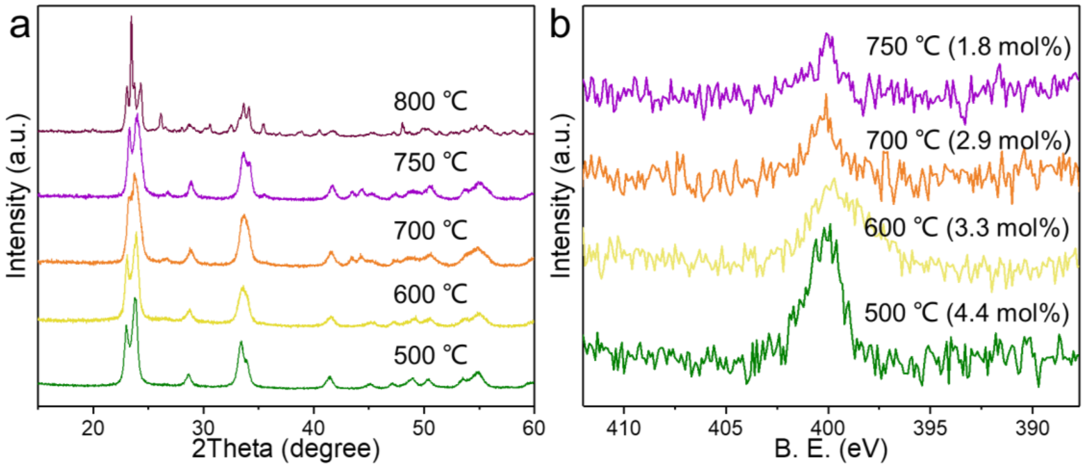

Supplementary Fig. 40. (a) XRD patterns of mN-WO_3 after the thermal treatment at 500, 600, 700, 750 and 800 °C in N_2. (b) XPS of mN-WO_3 after the thermal treatment at 500, 600, 700 and 750 °C in N_2 and followed at 400 °C in air. These results reveal that with the increase of calcination temperature in N_2, the content of nitrogen gradually declines but still exists, and the XRD patterns remain unchanged until the formation of WO_2 at 800 °C.

15. Spectrum characterization of mN-WO_3

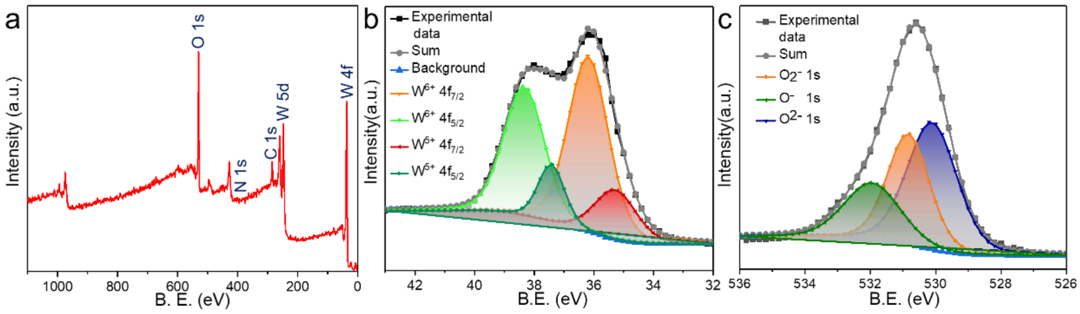

Supplementary Fig. 41. XPS of the samples (a) full spectrum, (b) W 4*f* and (c) O 1*s* core level peak regions of the mN-WO_3.

X-ray photoelectron spectroscopy (XPS) was employed to gain information about the valance states of the N-WO_3. The W 4*f* and O 1*s* core-level peaks are shown in Figure S39b and c. The N 1*s* core-level peak is shown in Figure 4f. The peaks at 36.2 and 38.3 eV correspond to W^{6+} $4f_{7/2}$ and W^{6+} $4f_{5/2}$, respectively. The peaks at 35.3 and 37.4 eV correspond to W^{5+} $4f_{7/2}$ and W^{5+} $4f_{5/2}$, respectively, implying abundant local distortion and variation of the coordination mode for the W-O bond exist in the structure. The spectrum of O 1*s* discloses the existence of dissociative O^- (532.0 eV), molecular-type adsorbate O_2^- (530.8 eV) and lattice O^{2-} (530.1 eV). The O^- and O_2^- can be regarded as the adsorbed oxygen species.

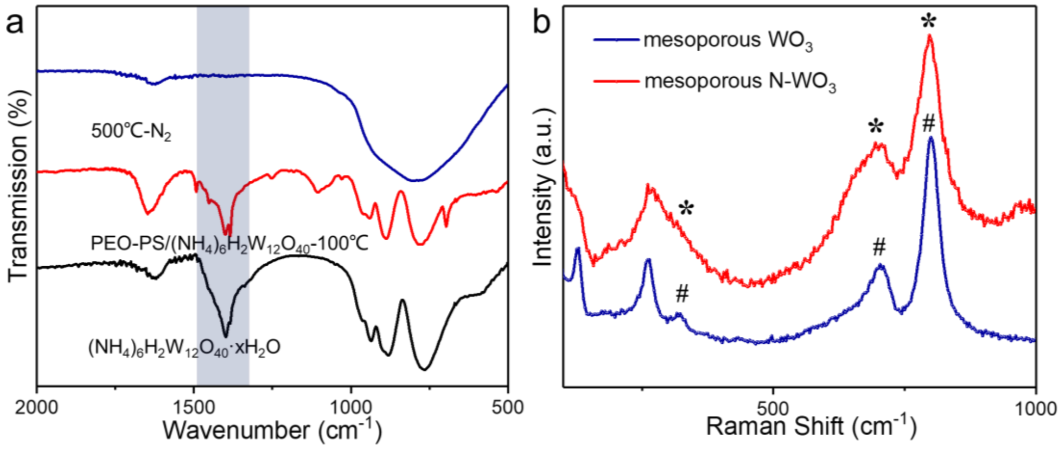

Supplementary Fig. 42. (a) FTIR of $(NH_4)_6H_2W_{12}O_{40}·xH_2O$, PEO-*b*-PS/$(NH_4)_6H_2W_{12}O_{40}$ composites after sequential thermal treatment at 100 and 500 °C in N_2. (b) Raman spectra of mN-WO_3 synthesized by using AMT as tungsten precursor (red curve) and mWO_3 synthesized by using WCl_6 as tungsten precursor (blue curve).

The Fourier transform infrared spectra (FTIR) of $(NH_4)_6H_2W_{12}O_{40}·xH_2O$, PEO-*b*-PS/$(NH_4)_6H_2W_{12}O_{40}$ and N-WO_3 are shown in **Supplementary Fig. 42**a. The vibration at 1400 cm^{-1} is attributed to NH_4^+ counter ions. The three bands at 937, 881 and 768 cm^{-1} are typical modes for metatungstate clusters with a Keggin-type structure and can be attributed to $v(W\text{-}O_t)$, $v(W\text{-}O_b\text{-}W)$ and $v(W\text{-}O_c\text{-}W)$. After thermal treatment at 500 °C in N_2, all the above peaks disappear, indicative of the complete decomposition of NH_4^+ ions and Keggin-type metatungstate clusters. A new broad peak at 804 cm^{-1} appears, corresponding to typical WO_3 materials. Raman spectra of mN-WO_3 and mWO_3 are shown in Supplementary Fig. 42b. mN-WO_3 with more vibration peaks is assigned to acentric *ε*-phase (space group: *Pc*), while mWO_3 is assigned to *γ*-WO_3 (space group: $P2_1/n$), both of which are consistent with the results of XRD patterns (Figure 4g).

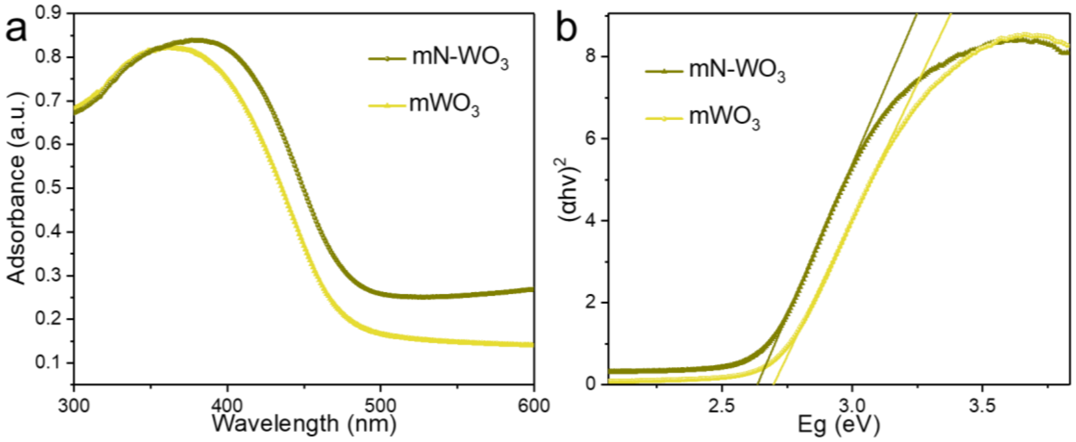

Supplementary Fig. 43. (a) UV-vis diffuse reflectance spectra of mN-WO_3 synthesized by using AMT as tungsten precursor (green curve) and mWO_3 synthesized by using WCl_6 as tungsten precursor (yellow curve). (b) Kubelka-Munk function curve plotted against photon energy for mN-WO_3 (green curve) and mWO_3 (yellow curve). The results reveal that the doping of nitrogen causes the reduction of band gap from 2.70 eV to 2.63 eV.

16. X-ray absorption fine structure data of mN-WO_3

The W L_3-edge XAFS for pure mWO_3 and mN-WO_3 were obtained from the Shanghai Synchrotron Radiation Facility, and all the data were recorded with a typical transmission mode. The obtained XAFS data was processed in Athena (version 0.9.26) for background, pre-edge line and post-edge line calibrations. Then Fourier transformed fitting was carried out in Artemis (version 0.9.26). The k^3 weighting, k-range of 3-12 $Å^{-1}$ and R range of 1-3 Å were used for the fitting. The four parameters, coordination number, bond length, Debye-Waller factor and E_0 shift (CN, R, σ^2, ΔE_0) were fitted without fixing, constraining, or correlating any parameter.

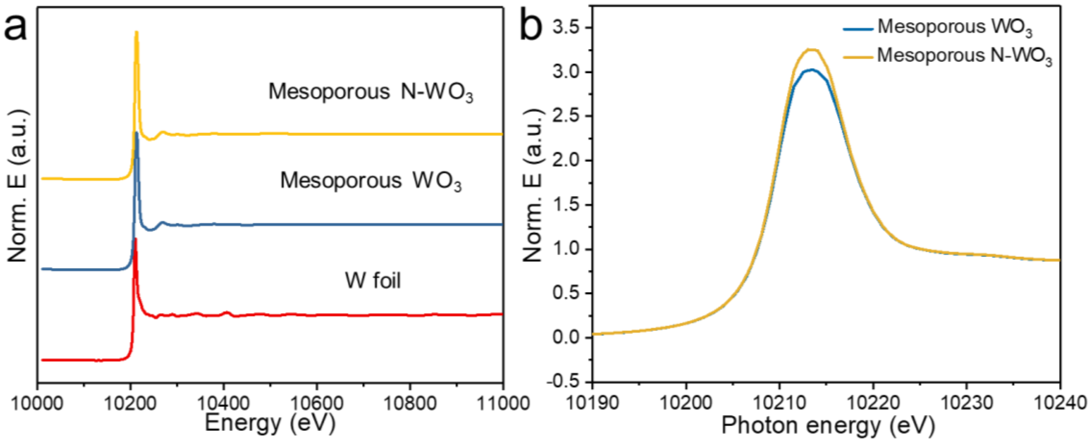

Supplementary Fig. 44. (a) The normalized X-ray absorption near-edge structure (XANES) spectra of W foil (red curve), mesoporous WO_3 (blue curve) and mesoporous N-WO_3 (yellow curve). (b) W L3-edge XANES spectra of mesoporous N-WO_3 (yellow curve) and mesoporous WO_3 (blue curve).

Supplementary Table 5. Extended X-ray absorption fine structure fitting parameters at the W L3-edge various samples (S_0^2 = 0.85).

Sample	Path	C.N.	R (Å)	$\sigma^2 \times 10^3$ ($Å^2$)	ΔE (eV)	R factor
W foil	W-W	8*	2.73±0.01	2.6±0.5	6.4±0.9	0.001
	W-W	6*	3.16±0.01	0.9±0.7	9.1±2.5	
WO_3	W-O	3.8±0.5	1.78±0.01	3.8±0.7	6.4±2.7	0.006
	W-O	2.1±0.7	2.07±0.01	16.0±5.7		
N-WO_3	W-O	2.6±0.4	1.78±0.01	1.6±0.6	8.3±2.2	0.005
	W-O	2.5±1.1	2.07±0.01	24.3±0.9		

17. DFT calculations and acetone sensing tests of mN-WO_3 based gas sensor

The first-principles were employed to perform spin-polarization DFT calculations[13,14] within the generalized gradient approximation (GGA) using the Perdew-Burke-Ernzerhof (PBE) formulation[15]. The projected augmented wave (PAW) potentials were chosen to describe the ionic cores by taking valence electrons into account using a plane wave basis set with a kinetic energy cutoff of 520 eV[16,17]. Partial occupancies of the Kohn−Sham orbitals were allowed using the Gaussian smearing method and a width of 0.01 eV. The electronic energy was considered self-consistent when the energy change was smaller than 10^{-6} eV. A geometry optimization was considered convergent when the energy change was smaller than 0.03 eV $Å^{-1}$. The dipole correction was used in this study. The vacuum spacing in a direction perpendicular to the plane of the structure is 15 Å. The Brillouin zone integration was performed using 3×3×1 Monkhorst-Pack k-point sampling for a structure. In our work, the WO_3 surfaces were established using the WO_3 of 2×2×2 supercell structure. Finally, the adsorption energies (E_{ads}) were calculated as $E_{ads} = E_{ad/sub} - E_{ad} - E_{sub}$, where $E_{ad/sub}$, E_{ad}, and E_{sub} are the total energies of the optimized adsorbate/substrate system, the adsorbate in the structure, and the clean substrate, respectively.

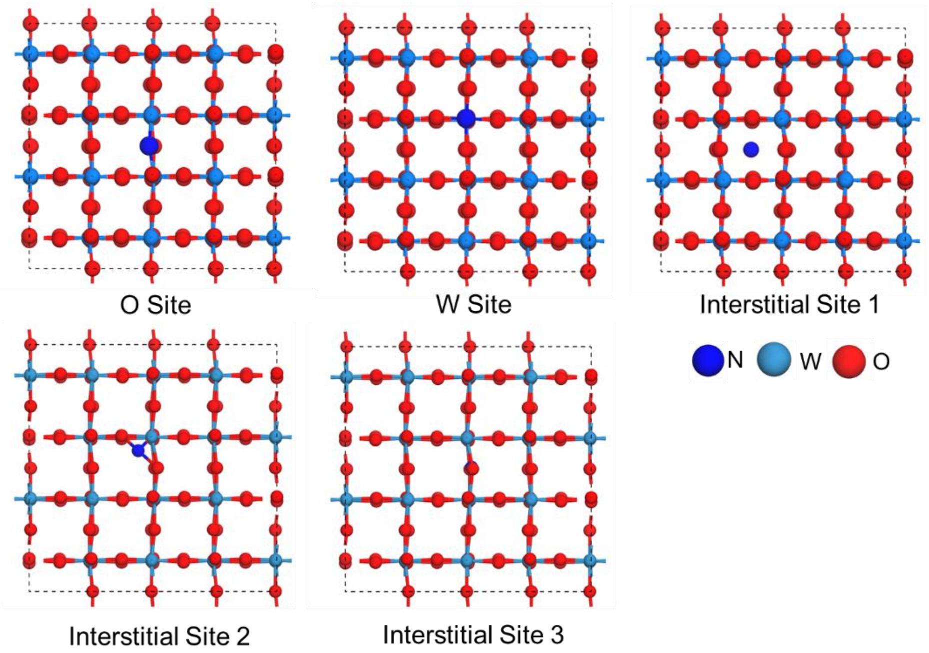

Supplementary Fig. 45. The optimized geometrical structures of nitrogen doping type in WO_3. O site means nitrogen atom replaces the location of oxygen atom. W site means nitrogen atom replaces the location of tungsten atom.

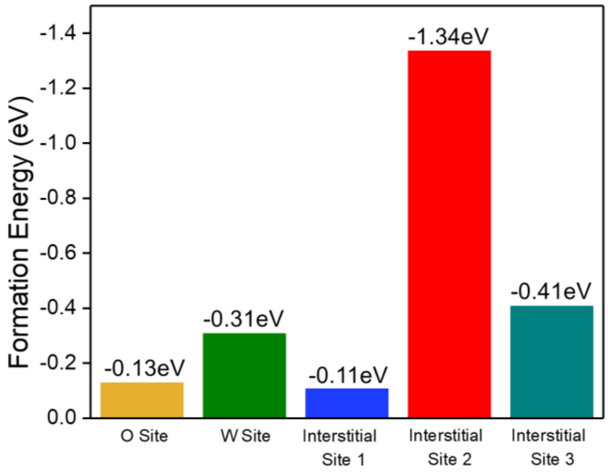

Supplementary Fig. 46. The comparison of formation energies of nitrogen doping in WO_3.

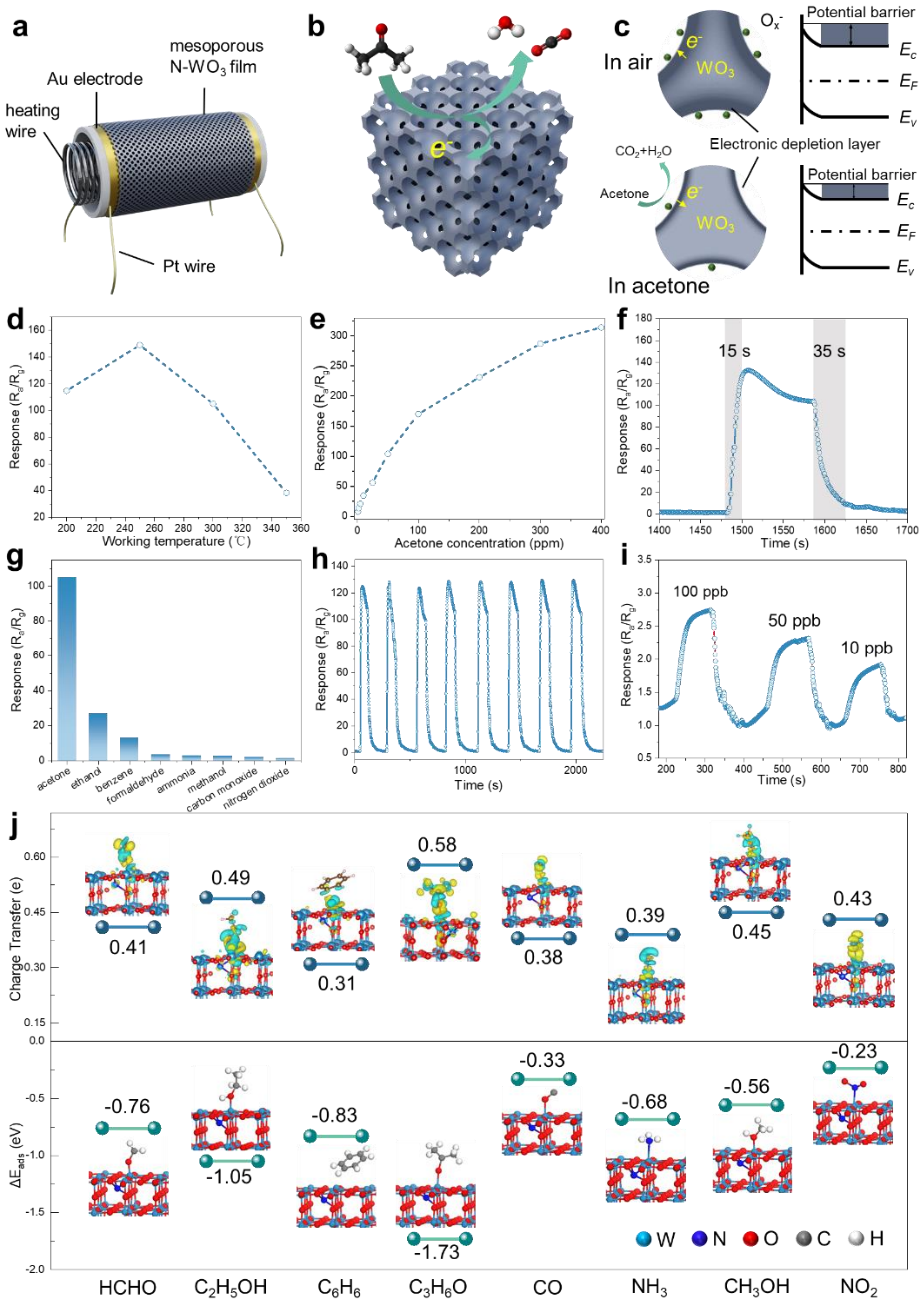

Supplementary Fig. 47. Gas sensing performances of the mN-WO3. (a) Sketch of the structure of a side-heated gas sensor on mN-WO$_3$. (**b** and **c**) Diagrams of the reaction on the surface of mN-WO$_3$ (b) and electron depletion layer model (c) to illustrate the interaction between mN-WO$_3$ and acetone. **(d)** Responses of the mN-WO$_3$ sensor to 50 ppm acetone at a temperature range of 200-350 °C. **(e)** Response value ($S = R_a/R_g$, which is defined as the ratio of the sensor resistance in air (R_a) to resistance in tested gas (R_g) for reducing gases) of the mN-WO$_3$ sensor to acetone at 300 °C. **(f)** Response-recovery curve of the mN-WO$_3$ sensor to 50 ppm acetone at 300 °C. **(g)** Responses of the mN-WO$_3$ sensor to different gases of 50 ppm. **(h)** Repeating response-recovery curve of the mN-WO$_3$ sensor to 50 ppm acetone at 300 °C to illustrate the cycling stability. **(i)** Response-recovery curve of the mN-WO$_3$

sensor in ppb-level acetone sensor. **(j)** Comparison of the adsorption energies and charge density difference of eight gases on the (020) plane of N-WO_3. The blue and yellow lobes represent the charge depletion and accumulation for gas adsorption and reduction. Lower adsorption energy denotes stronger adsorption.

The gas sensor platform and sensing mechanism are shown in Supplementary Fig. 47a-c. To optimize the working temperature, a series of tests was carried out toward 50 ppm of acetone at 200-350 °C and the activity reached its maximum at 250 °C (Supplementary Fig. 47d). However, the recovery of the resistance at 250 °C is slow (several minutes). To balance the high activity and fast response-recovery dynamics, 300 °C was chosen as the optimal working temperature. The mN-WO_3 gas sensors show a rapid response and good reversibility from 7.8 to 314 within 1-400 ppm (Supplementary Fig. 47e). In 50 ppm acetone vapor, the mN-WO_3 sensors display response of 15 s and recovery of 35 s, indicative of fast response-recovery dynamics (Supplementary Fig. 47f).

For comparison, the gas-sensing performance of aforementioned mWO_3 was investigated, while its response rate is much slower and response value is much lower than mN-WO_3 (Supplementary Fig. 47g). The mWO_3 with polycrystalline walls has large amounts of grain boundaries, which may hinder the rapid migration of electrons. By contrast, the iso-oriented crystalline walls of mN-WO_3 facilitate the carriers' migration and lead to a faster response rate. Besides, the nitrogen doping enhances the polarity of mN-WO_3, which causes the relatively strong interactions between acetone and mN-WO_3, and thus further accelerate the response and lead to higher response value due to the enhanced acetone adsorption and charge transfer.

The response value of the sensors to 50 ppm acetone is 103.7, at least four times

higher than the seven interference gases under the same conditions, indicating the good selectivity of mN-WO_3 sensors (Supplementary Fig. 47g). Moreover, the mN-WO_3 sensors have good responses to low concentrations of acetone with ppb-level (S = 1.9 for 10 ppb), indicative of great potential for detection of trace acetone (the threshold value for the biomarker of acetone in diabetic breath is 1.1 ppm, Supplementary Fig. 47i). After gas sensing tests and storing for one year, the mesostructures of mN-WO_3 deposited on alumina tubes can be basically retained and only accompanied by slight collapse of pore walls, indicating the good structure stability of mN-WO_3 **(Supplementary Fig. 48)**. DFT calculations indicate that high selectivity toward acetone of mN-WO_3-based gas sensors originates from the favorable adsorption and significant electronic transfer (Supplementary Fig. 47j, **49-51**). Compared to previously reported acetone sensors based on other materials, the mN-WO_3 sensors show outstanding comprehensive sensing performances (**Supplementary Table 6**).

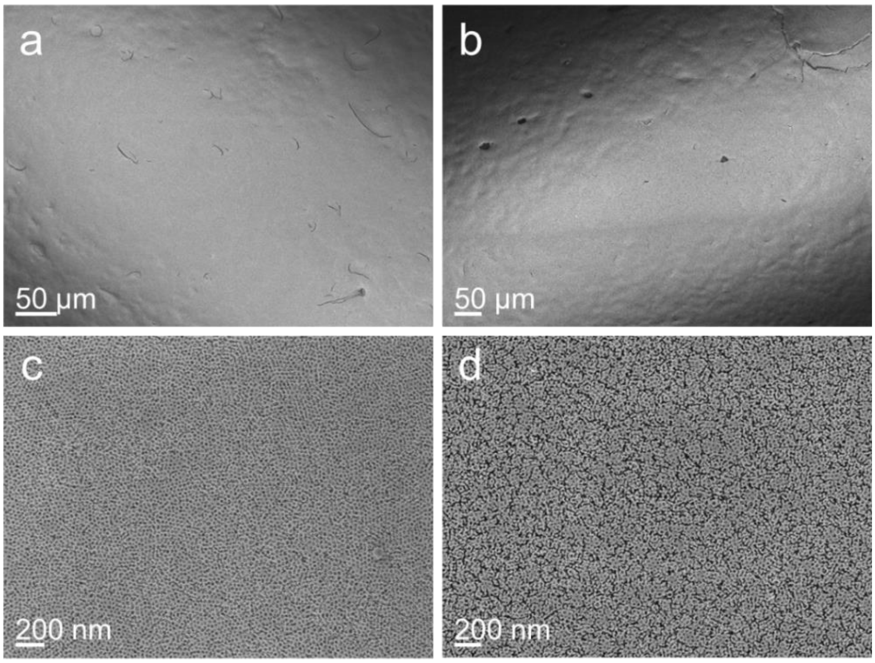

Supplementary Fig. 48. (a, c) SEM images of mN-WO_3 deposited on alumina tubes before gas sensing tests. (b, d) Same bath of sensors after long-term gas sensing tests and storing for one year.

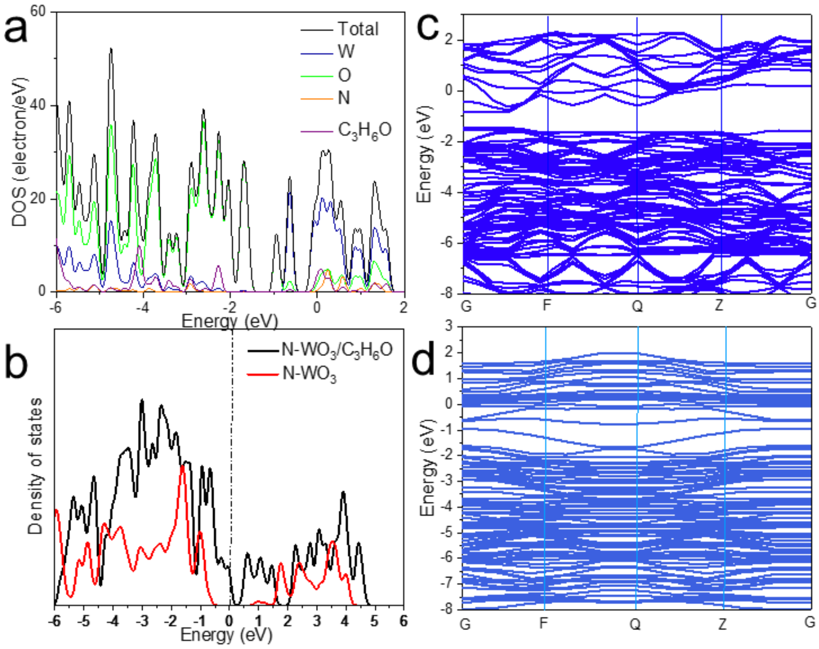

Supplementary Fig. 49. (a) The DOS diagrams of N-WO_3+C_3H_6O. (b) The DOS diagrams of N-WO_3 before and after acetone adsorption. (c and d) The calculated band structures of the N-WO_3 before and after acetone adsorption.

To elucidate the accurate electronic transfer associated with the gas sensing process, extensive theoretical investigations using the DFT model were calculated. The (020) plane of monoclinic WO_3 with nitrogen-interstitial doping (interstitial site 2 in Supplementary Fig. 42) was chosen as the surface. The DOS of the N-WO_3/C_3H_6O displays a new energy level in the conduction band (**Supplementary Fig. 49** a and b) and the band gap is narrowed (Supplementary Fig. 49 c and d) due to the strong bonding adsorption of C_3H_6O on the N-WO_3 surface. The results consistently demonstrate that the C_3H_6O adsorption changes the surface energy level and the electronic structure of the N-WO_3.

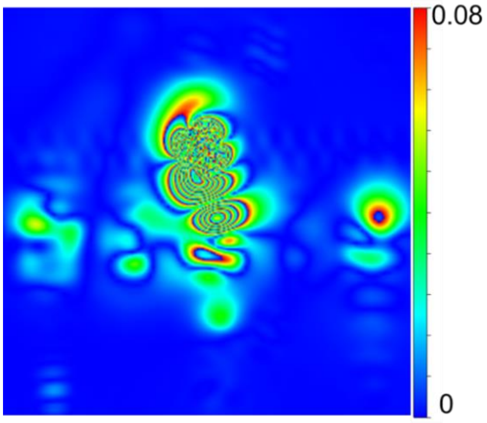

Supplementary Fig. 50. Charge distribution in a 2D plane of the N-WO_3 after C_3H_6O adsorption.

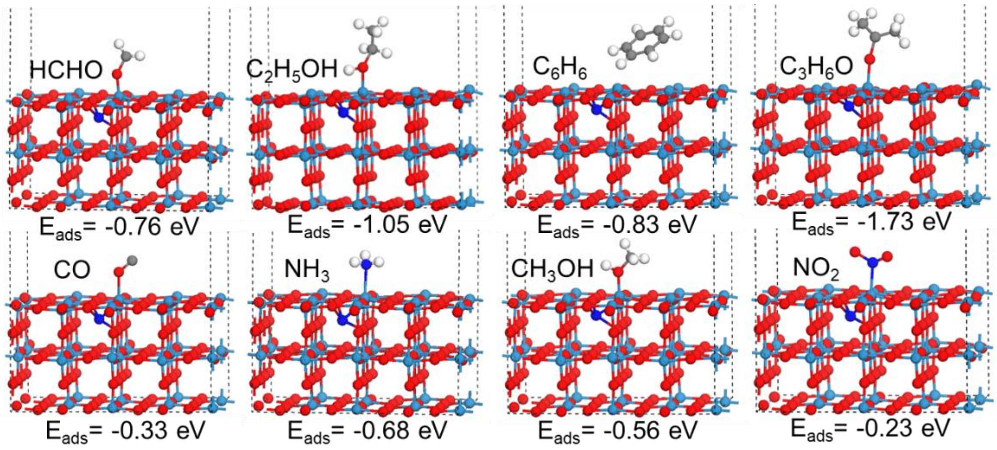

Supplementary Fig. 51. The analysis of the adsorption structure with geometric binding configuration for the model of eight gases on the (020) plane of N-WO_3.

The adsorption configurations of the different gases on the N-WO_3 were optimized and no obvious changes in configurations were observed after the adsorption of gases molecules onto the N-WO_3 (**Supplementary Fig. 51**). The sensor exhibits the highest acetone (C_3H_6O) adsorption energies of -1.73 eV among all the calculated interference gases, which is consistent with the experimental results displayed in Supplementary Fig. 47g. These results confirm the high selectivity of the N-WO_3 for acetone molecule. All the E_{ads} values are less than 2.0 eV, indicating the physically adsorbed process of all the gases on the N-WO_3 nanosheets.

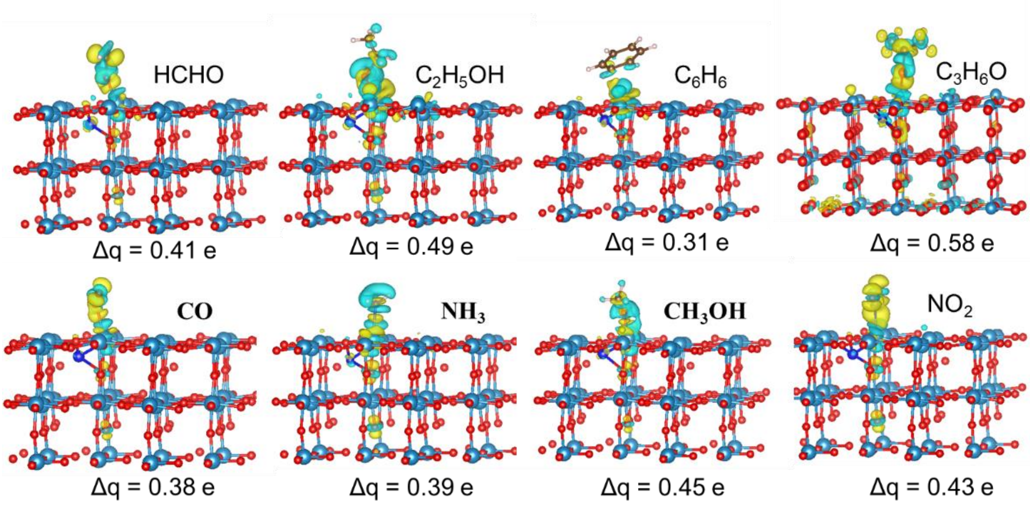

Supplementary Fig. 52. Calculated charge transfer for different gases on the (020) plane of N-WO_3.

Supplementary Table 6. Comparison about the acetone gas sensing performance of the reported materials and mN-WO_3 in this work

**Sensing materials**	**Concentration [ppm]**	**Response value**	**Temperature [°C]**	**Response/ Recovery time [s]**	**References**
Mesoporous WO_3	50	~38	250	N/A	*Angew. Chem. Int. Ed.* **2014**, *53*, 9035-9040.
Mesoporous ZnO-SiO_2	50	33.4	240	13/-	*Chem. Mater.* **2019**, *31*, 8112-8120.
Mesoporous Al_2O_3/WO_3	10	~3.5	250	16/49	*J. Mater. Chem. A* **2019**, *7*, 21874.
Mesoporous WO_3/TiO_2	50	14	290	3/5	*Chin. Chem. Lett.* **2020**, *31*, 1119-1123.
Mesoporous Au-decorated In_2O_3	100	19.01	250	25/31	*J. Mater. Chem. C* **2020**, *8*, 78.
TiO_2@PW_{12}@ Fe_2O_3	100	14.81	360	1/25	*Sens. Actuators, B* **2023**, *378*, 133088.
$W_{18}O_{49}$ nanowires	50	48.6	280	11/13	*ACS Appl. Mater. Interfaces* **2020**, *12*, 3755-3763.
$W_{18}O_{49}/Ti_3C_2T_x$	20	11.6	300	4.6/18.2	*Sens. Actuators, B* **2020**, *304*, 127274.
Daisy-like *h*-WO_3	50	36.7	260	5/7	*Sens. Actuators, B* **2021**, *329*, 129188.
In_2O_3-WO_3 microspheres	100	55	280	N/A	*Sens. Actuators, B* **2022**, *361*, 131705.
Pt-loaded WO_3-SnO_2 NTs	50	63.8	275	N/A	*Sens. Actuators, B* **2020**, *306*, 127575.
MOF-derived Co_3O_4	50	27.6	140	70/64	*Sens. Actuators, B* **2023**, *376*, 132973.
Hollow hexagram-shaped Co_3O_4	100	23.32	150	48/5	*Chem. Eng. J.* **2023**, *471*, 144297.
Oxygen-plasma-treated ZnO	100	~125	250	75/125	*ACS Appl. Mater. Interfaces* **2020**, *12*, 23084-23093.
Pt-modified $BiVO_4$ nanosheets	100	12.5	300	2/61	*Sens. Actuators, B* **2023**, *389*, 133853.
$LaFe_{0.99}P_{0.01}O_{3-\delta}$ nanosheets	100	30	180	N/A	*Chem. Eng. J.* **2022**, *431*, 134280.
Okra-shaped porous Co_3O_4	100	35.2	150	109/42	*ACS Appl. Electron. Mater.* **2021**, *3*, 3400-3410.

α-Bi_2O_3 nanowires	10	2.93	350	N/A	*Sens. Actuators, B* **2021**, *346*, 130432.
ZnO/ZnSe	100	80.8	325	51/11	*J. Alloy. Compd.* **2022**, *906*, 164316.
$Bi_{0.9}La_{0.1}FeO_3$	100	~45	260	N/A	*Sens. Actuators, B* **2020**, *313*, 128060.
**Mesoporous N-WO_3**	**50**	**103.7**	**300**	**15/35**	**This work**

N/A: Not applicable. The gas response of the sensor in this study is defined as $S = R_a/R_g$.

18. Co-assembly of AMT and other POMs

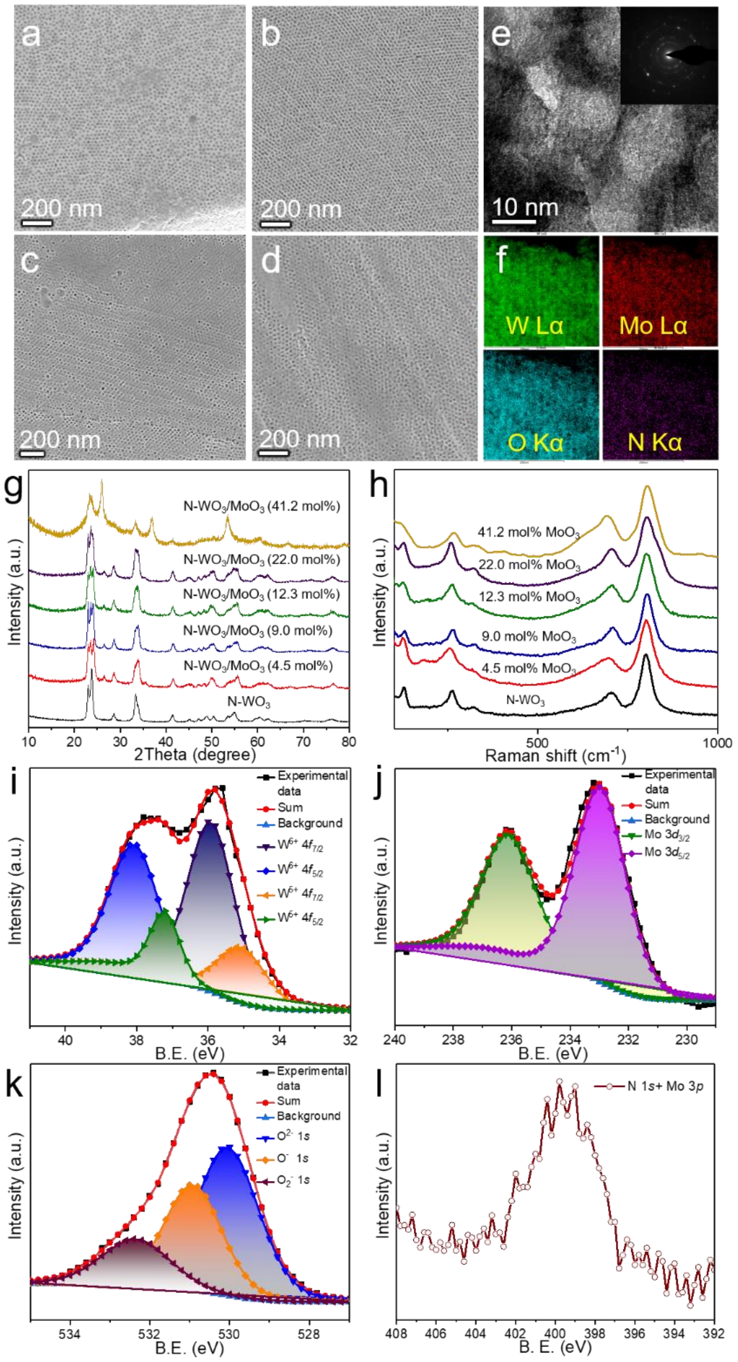

Supplementary Fig. 53. Co-assembly of AMT and $(NH_4)_6Mo_7O_{24}$. (a-d) SEM images of mesoporous N-WO_3/MoO_3 composites with different ratios of constituents: (a) 4.5 mol% MoO_3, (b) 9 mol% MoO_3, (c) 12.3 mol% MoO_3 and (d) 41.2 mol% MoO_3. (e) HRTEM image of mesoporous N-WO_3/MoO_3 (4.5 mol%). The inset in (e) is the selected-area electron diffraction pattern revealing the crystalline nature. (f) Element mapping of the mN-WO_3/MoO_3 (4.5 mol%). (g) XRD patterns and (h) Raman spectra of the mesoporous N-WO_3/MoO_3 composites with different ratios of constituents. XPS showing the (i) W 4*f*, (j) Mo 3*d*, (k) O 1*s* and (l) N 1*s*+ Mo 3*p* core level peak regions of the N-WO_3/MoO_3 (4.5 mol%).

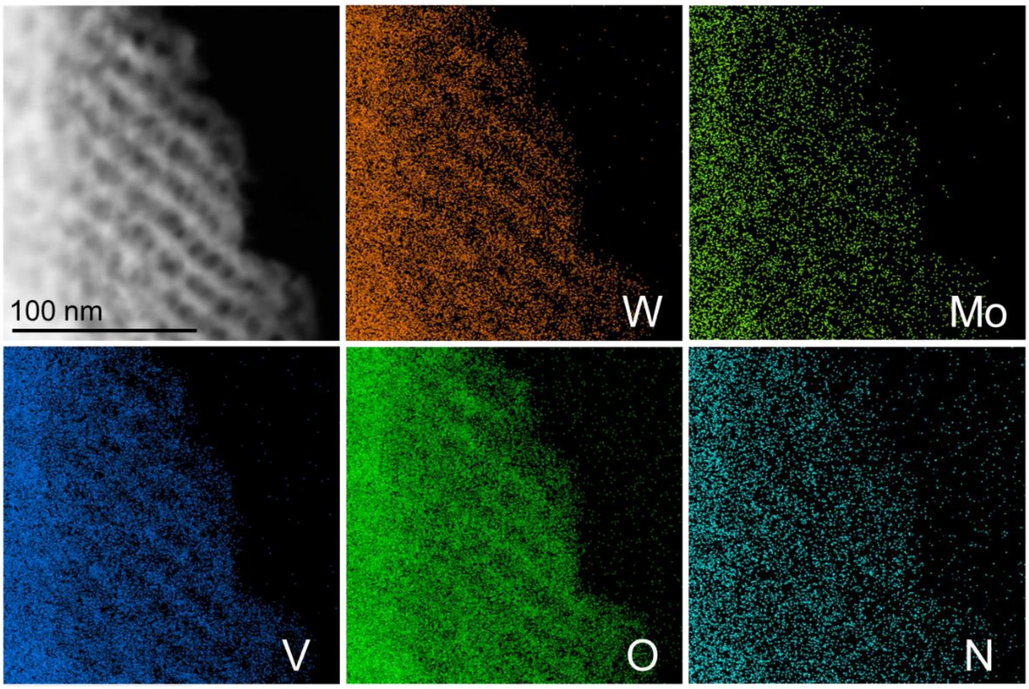

Supplementary Fig. 54. Co-assembly of AMT, $(NH_4)_6Mo_7O_{24}$ and $(NH_4)_4V_{10}O_{24}$. Element mapping shows the uniform distribution of W, Mo, V, O and N throughout the mMO composites, indicative of homogeneous assembly of these POMs.

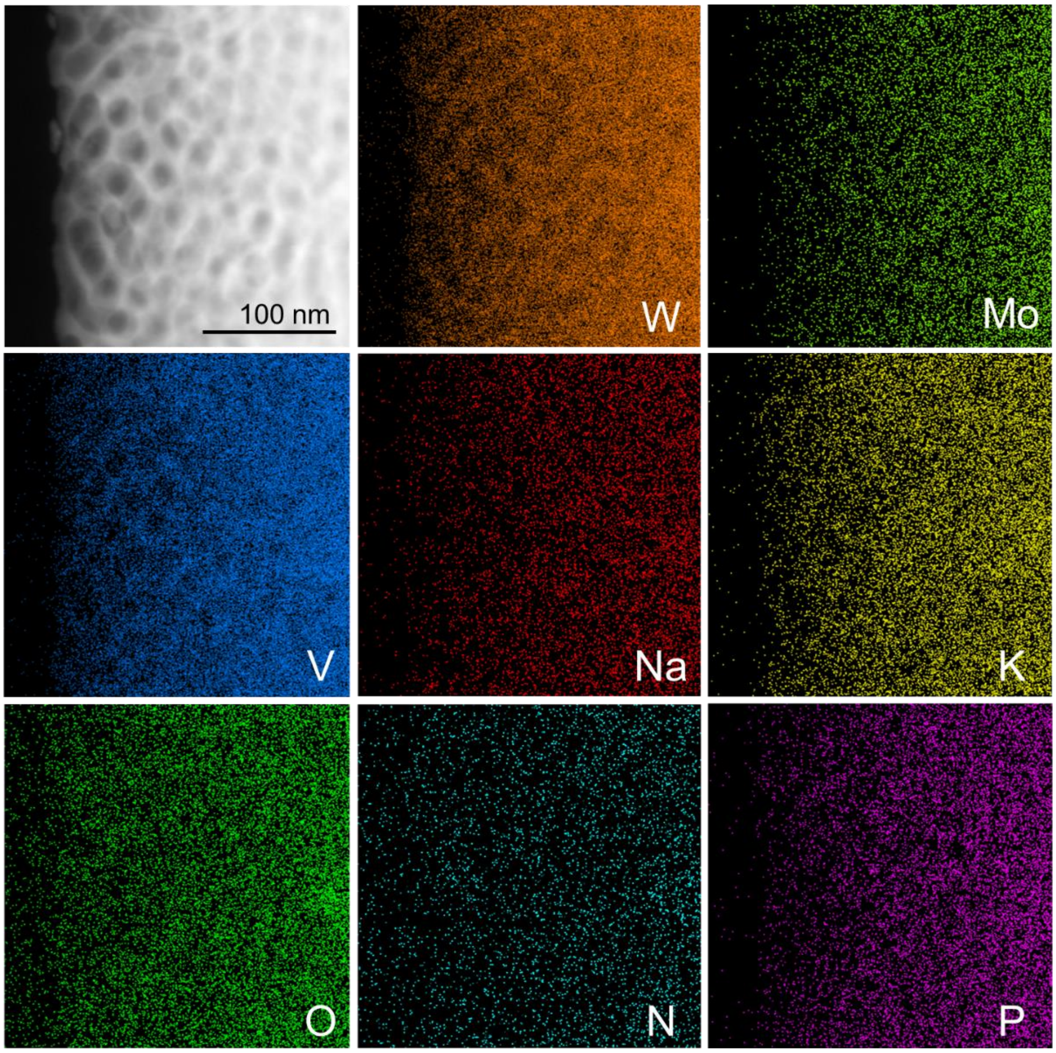

Supplementary Fig. 55. Co-assembly of nine kinds of non-acidic POMs, including AMT, $(NH_4)_6Mo_7O_{24}$, $(NH_4)_4V_{10}O_{24}$, $Na_6H_2W_{12}O_{40}$, $Na_3PW_{12}O_{40}$, $Na_4V_{10}O_{28}$, $K_4V_{10}O_{28}$, $Li_4V_{10}O_{28}$ and $K_3Mo_6O_{19}$. Element mapping shows the uniform distribution of W, Mo, V, Na, K, O, N and P throughout the mMO composites, indicative of homogeneous assembly of these POMs. Li cannot be detected by EDS due to its low atomic number.

The SPEA method can be used to assemble multiple kinds of POMs to construct heteroatoms doped or composite mMOs via one-pot synthesis. For example, the co-assembly of AMT, $(NH_4)_6Mo_7O_{24}$ and PEO-*b*-PS, can be used to synthesize mesoporous N-WO_3/MoO_3 composites. The synthesis process follows the same method as that for mN-WO_3 except that AMT and $(NH_4)_6Mo_7O_{24}$ were used as the

precursors. The ratio of constituents can be tuned in a wide range. SEM images confirm that the mesostructure remains ordered as the increase of MoO_3 content (**Supplementary Fig. 53**a-d). Element mapping shows the uniform distribution of W, Mo, O and N throughout the mN-WO_3/MoO_3, indicative of homogeneous mixing of the N-WO_3 and N-MoO_3 (Figure S53f). XRD patterns of the mN-WO_3/MoO_3 samples exhibit well-resolved similar diffraction peaks within 0-22 mol% MoO_3 content. The diffraction peaks of MoO_3 occur in mN-WO_3/MoO_3 (41.2 mol%), indicating the high content and large grains of MoO_3 phase (Supplementary Fig. 53g). With the increase of MoO_3 content, the absorption peaks of Raman spectra show a blueshift induced by the Mo-O lattice vibrations, which confirms that the MoO_3 phase is chemically mixed with the WO_3 phase at the atomic level (Supplementary Fig. 53h). These results confirm that the AMT and $(NH_4)_6Mo_7O_{24}$, as well as the obtained N-WO_3 and N-MoO_3, are homogeneously mixed during the co-assembly and calcination process. Following the similar method, this assembly system can be extended to three kinds of POMs co-assembly (**Supplementary Fig. 54**), and even successfully applied to nine kinds of POMs co-assembly (**Supplementary Fig. 55**).

19. Co-assembly of AMT and molecular metal salts

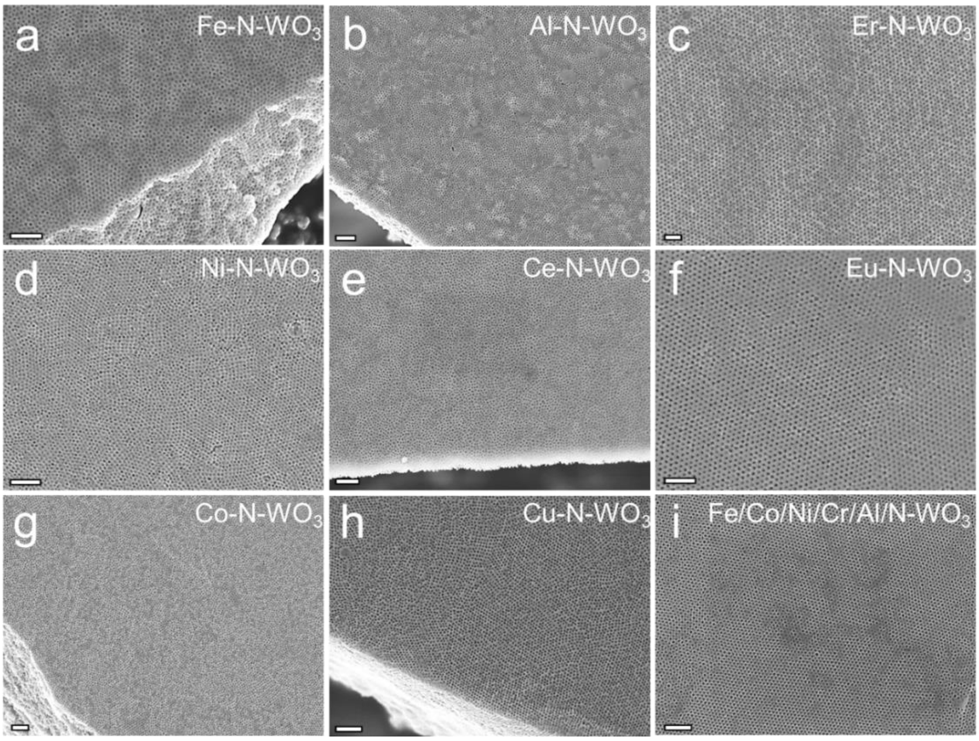

Supplementary Fig. 56. (a-i) SEM images of mesoporous Fe-N-WO_3 (a), Al-N-WO_3 (b), Er-N-WO_3 (c), Ni-N-WO_3 (d), Ce-N-WO_3 (e), Eu-N-WO_3 (f), Co-N-WO_3 (g), Cu-N-WO_3 (h) and Fe/Co/Ni/Cr/Al/N-WO_3 (i) synthesized by co-assembly of AMT with molecular metal salts, respectively. The scale bars are 200 nm.

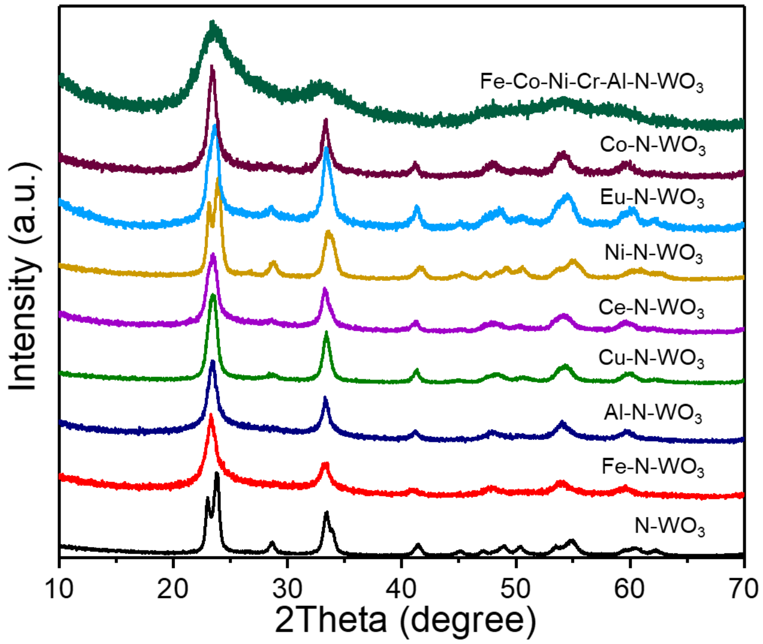

Supplementary Fig. 57. XRD patterns of M-N-WO_3 (M = Fe, Al, Er, Ni, Ce, Eu, Co, Cu, Cr, *etc*.) synthesized by co-assembly of AMT and molecular metal salts.

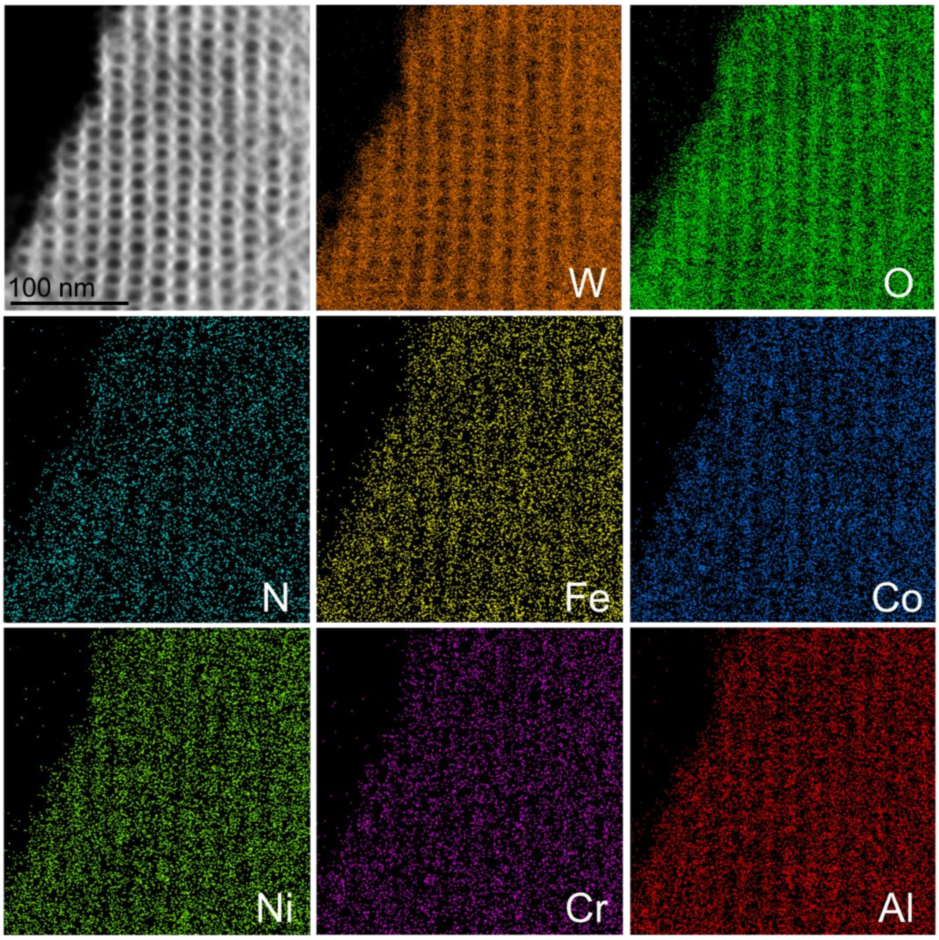

Supplementary Fig. 58. Element mapping of Fe-Co-Ni-Cr-Al-N-WO_3. The uniform distribution of these elements indicates that the homogeneous doping of the Fe, Co, Ni, Cr and Al in the mN-WO_3.

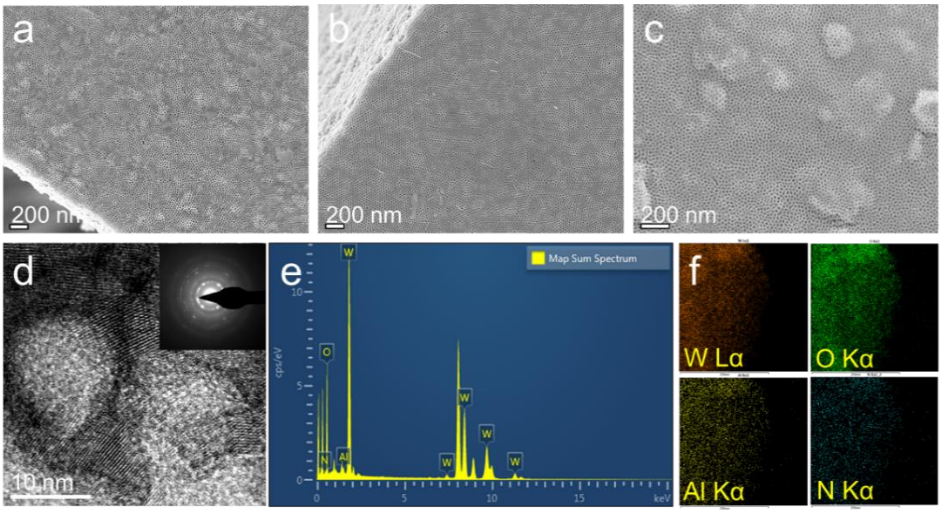

Supplementary Fig. 59. The co-assembly of AMT and $AlCl_3$. (a-c) SEM images of mesoporous N-WO_3/Al_2O_3 composites with different ratios of constituents: (a) 5.8 mol% Al_2O_3, (b) 11 mol% Al_2O_3 and (c) 20 mol% Al_2O_3. (D) HRTEM image of N-

WO_3/Al_2O_3 (5.8 mol%). (e) Energy dispersive spectrum and (f) element mapping show the uniform distribution of W, Al, O and N throughout the mN-WO_3/Al_2O_3, indicative of homogeneous mixing of the WO_3 and Al_2O_3.

The SPEA method can be used to co-assemble POMs with molecular metal salts to construct heteroatoms doped, noble metal nanoparticles loaded and composite mMOs via one-pot synthesis. As shown in **Supplementary Fig. 56**, nine kinds of metal and nitrogen atoms co-doped mesoporous WO_3 (M-N-WO_3) were synthesized following the same method as that of the mN-WO_3 except that AMT and metal salts were used as the precursors. The used metal salts can be the single constituent or the combinations of $FeCl_3$, $AlCl_3$, $Cu(CH_3COO)_2$, $NiCl_2$, $CeCl_3$, $Co(CH_3COO)_2$, $Eu(NO_3)_2$, $Er(NO_3)_2$ and $Cr(NO_3)_3$ and the mass ratio of AMT: metal salts keeps 30:1. XRD patterns of M-N-WO_3 exhibit well-resolved similar diffraction peaks with N-WO_3, indicative of a uniform mixing or doping of metal atoms in N-WO_3 (**Supplementary Fig. 57**). The ratio of constituents can be tuned in a wide range. Taking the co-assembly of AMT and $AlCl_3$ as an example, the obtained mN-WO_3/Al_2O_3 can retain ordered mesostructure with the increase of Al_2O_3 content (**Supplementary Fig. 59**).

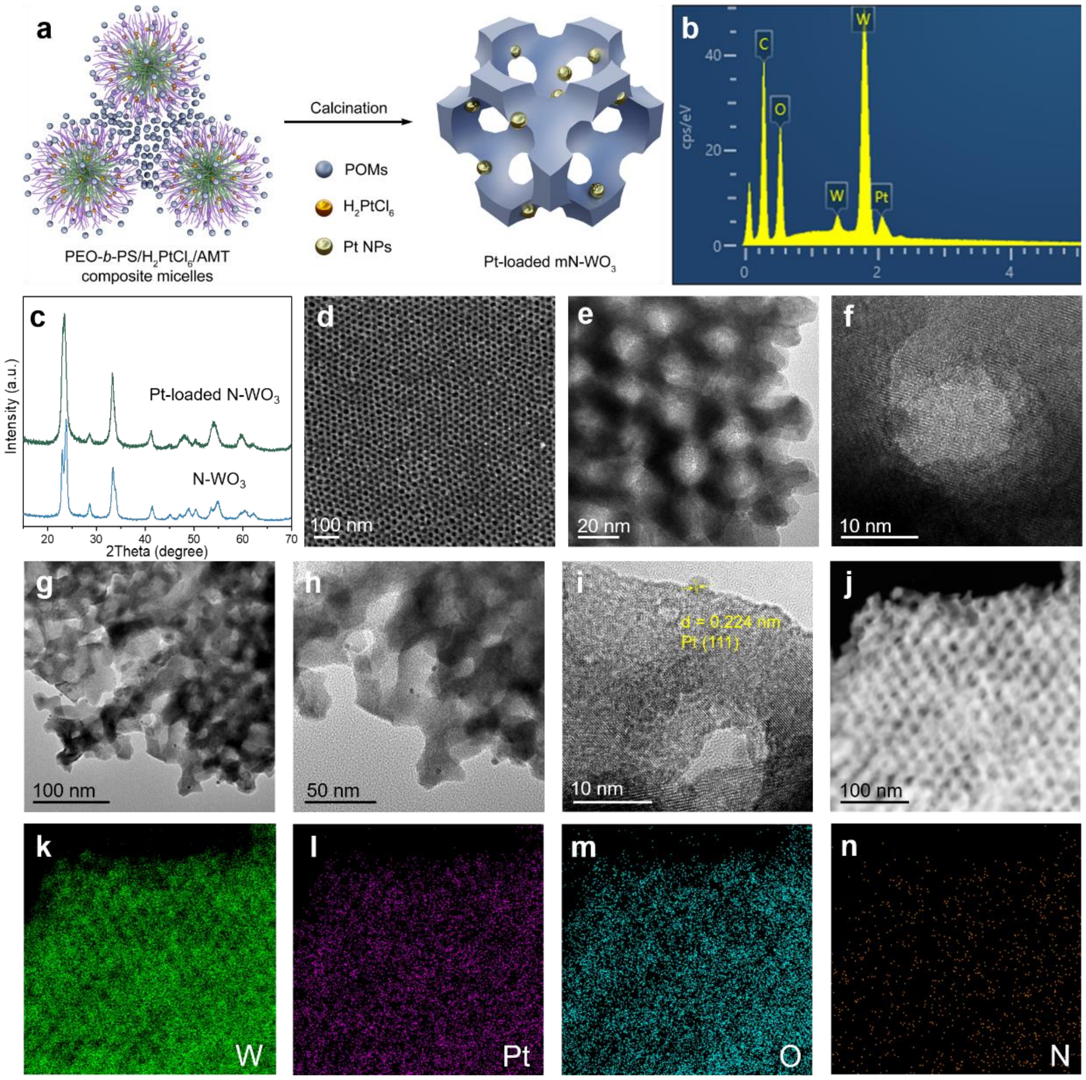

Supplementary Fig. 60. The co-assembly of AMT and H_2PtCl_6. (a) Scheme illustration of the synthesis of Pt-loaded mN-WO_3. (b) Energy dispersive spectrum and (c) XRD patterns of Pt-loaded mN-WO_3 (4.6 mol% Pt, green curve) and N-WO_3 (blue curve). (d) SEM image, (e-i) TEM images and (j) HAADF-STEM image of Pt-loaded mN-WO_3. (k-n) Element mapping shows the uniform distribution of W, Pt, O and N throughout the Pt-loaded mN-WO_3 (4.6 mol% Pt), indicative of a homogenous Pt loading.

The direct co-assembly of AMT and H_2PtCl_6 can be used to achieve homogeneous loading of Pt nanoparticles within mN-WO_3, which is useful in heterogeneous catalysis. XRD patterns show similar diffraction peaks of mN-WO_3 and Pt-loaded mN-WO_3, indicative of the small size of loaded Pt nanoparticles. TEM images further confirm that the Pt nanoparticles possess ultrasmall grain size. Element

mapping shows the uniform distribution of W, Pt and O throughout the Pt-loaded mN-WO_3 (4.6 mol% Pt), indicative of the formation of highly dispersed Pt nanoparticles within mN-WO_3 (**Supplementary Fig. 60**).

Supplementary References

1. Frisch, M. J., et al. Gaussian 16, Revision A.03: Gaussian, Inc., Wallingford CT, (2016).
2. Zhao, Y. & Truhlar, D. G. The M06 suite of density functionals for main group thermochemistry, thermochemical kinetics, noncovalent interactions, excited states, and transition elements: two new functionals and systematic testing of four M06-class functionals and 12 other functionals. *Theor. Chem. Acc.* **120**, 215-241 (2008).
3. Lu, T. & Manzetti, S. Wavefunction and reactivity study of benzo [a] pyrene diol epoxide and its enantiomeric forms. *Struct. Chem.* **25**, 1521-1533 (2014).
4. Lu, T. & Chen, F. J. Multiwfn: A multifunctional wavefunction analyzer. *Comput. Chem.* **33**, 580-592 (2012).
5. Zhang, J. & Lu, T. Efficient evaluation of electrostatic potential with computerized optimized code. *Phys. Chem. Chem. Phys.* **23**, 20323-20328 (2021).
6. Humphrey, W., Dalke, A. & Schulten, K. VMD: visual: molecular dynamics. *J. Mol. Graphics* **14**, 33-38 (1996).
7. Abraham, M. J., et al. GROMACS: High performance molecular simulations through multi-level parallelism from laptops to supercomputers. *SoftwareX* **1-2**, 19−25 (2015).
8. Spoel, D. V. D. et al. GROMACS: Fast, flexible, and free. *J. Comput. Chem.* **26**, 1701-1718 (2005).
9. Wang, J., Wang, W., Kollman, P. A. & Case, D. A. Automatic atom type and bond

type perception in molecular mechanical calculations. *J. Mol. Graph. Model.* **25**, 247 (2006).

10. Cieplak, P., Cornell, W. D., Bayly, C .& Kollman, P. A. Application of the multimolecule and multiconformational RESP methodology to biopolymers: Charge derivation for DNA, RNA, and proteins. *J. Comput. Chem.* **16**, 1357-1377 (1995).
11. Bayly, C., Cornell, W. D. & Kollman, P. A. A well-behaved electrostatic potential based method using charge restraints for deriving atomic charges: the RESP model**.** *J. Phys. Chem.* **97**, 10269-10280 (1993).
12. Vasudevan, V. & Mushrif, S. H. Force field parameters for N,N-Dimethylformamide (DMF) revisited: Improved prediction of bulk properties and complete miscibility in water. *J. Mol. Liq.* **206**, 338-342 (2015).
13. Kresse, G. & Furthmüller, J. Efficiency of Ab-Initio Total Energy Calculations for Metals and Semiconductors Using a Plane-Wave Basis Set. *Comput. Mater. Sci.* **6**, 15-50 (1996).
14. Kresse, G. & Furthmüller, J. Efficient Iterative Schemes for Ab Initio Total-Energy Calculations Using a Plane-Wave Basis Set. *Phys. Rev. B* **54**, 11169-11186 (1996).
15. Perdew, J. P., Burke, K. & Ernzerhof, M. Generalized Gradient Approximation Made Simple. *Phys. Rev. Lett.* **77**, 3865-3868 (1996).
16. Kresse, G. & Joubert, D. From Ultrasoft Pseudopotentials to the Projector Augmented-Wave Method. *Phys. Rev. B* **59**, 1758-1775 (1999).

17. Blochl, P. E. Projector Augmented-Wave Method. *Phys. Rev. B* 50, 17953-17979 (1994).
